# A Reconfigurable Omnidirectional Triboelectric Whisker Sensor Array for Versatile Human–Machine–Environment Interaction

**DOI:** 10.1007/s40820-025-01930-x

**Published:** 2025-10-14

**Authors:** Weichen Wang, Jiaqi Zhu, Hongfa Zhao, Fei Yao, Yuzhu Zhang, Xiankuan Qian, Mingrui Shu, Zhigang Wu, Minyi Xu, Hongya Geng, Wenbo Ding, Juntian Qu

**Affiliations:** 1 Shenzhen International Graduate School, Tsinghua University, Shenzhen, 518055 People’s Republic of China; 2 State Key Laboratory of Digital Manufacturing Equipment and Technology, Huazhong University of Science and Technology, Wuhan, 430074 People’s Republic of China; 3 Institute of Biomedical and Health Engineering, Shenzhen International Graduate School, Tsinghua University, Shenzhen, 518055 People’s Republic of China; 4 Dalian Key Lab of Marine Micro/Nano Energy and Self-Powered System, Dalian Maritime University, Dalian, 116026 People’s Republic of China

**Keywords:** Reconfigurable sensor array, Interaction interface, Tactile perception, Omnidirectional sensor, Reversible anchoring

## Abstract

Developing effective, versatile, and high-precision sensing interfaces remains a crucial challenge in human–machine–environment interaction applications. Despite progress in interaction-oriented sensing skins, limitations remain in unit-level reconfiguration, multiaxial force and motion sensing, and robust operation across dynamically changing or irregular surfaces. Herein, we develop a reconfigurable omnidirectional triboelectric whisker sensor array (RO-TWSA) comprising multiple sensing units that integrate a triboelectric whisker structure (TWS) with an untethered hydro-sealing vacuum sucker (UHSVS), enabling reversibly portable deployment and omnidirectional perception across diverse surfaces. Using a simple dual-triangular electrode layout paired with MXene/silicone nanocomposite dielectric layer, the sensor unit achieves precise omnidirectional force and motion sensing with a detection threshold as low as 0.024 N and an angular resolution of 5°, while the UHSVS provides reliable and reversible multi-surface anchoring for the sensor units by involving a newly designed hydrogel combining high mechanical robustness and superior water absorption. Extensive experiments demonstrate the effectiveness of RO-TWSA across various interactive scenarios, including teleoperation, tactile diagnostics, and robotic autonomous exploration. Overall, RO-TWSA presents a versatile and high-resolution tactile interface, offering new avenues for intelligent perception and interaction in complex real-world environments.

**Supplementary Information:**

The online version contains supplementary material available at 10.1007/s40820-025-01930-x.

## Highlights

- Dual-triangular electrode layout with MXene/silicone nanocomposite achieves quite competitive omnidirectional force detection (threshold: 0.024 N) and angular resolution (5°) using only two electrodes.
- Based on a newly designed hydrogel combining high mechanical robustness and superior water absorption, the untethered hydro-sealing vacuum sucker can achieve robust and reversible anchoring on diverse surfaces with a compact structure, maintaining a consistently high anchoring force for more than 200 cycles with a single rehydration.
- The reconfigurable omnidirectional triboelectric whisker sensor array demonstrates exceptional performance in real-world applications, including teleoperation, adjustable robotic arm palpation, and robotic autonomous environmental exploration, validating its potential as a universal interface for dynamic human–machine–environment interactions.

**Supplementary Information:**

The online version contains supplementary material available at 10.1007/s40820-025-01930-x.

## Graphical abstract

**Figure:**
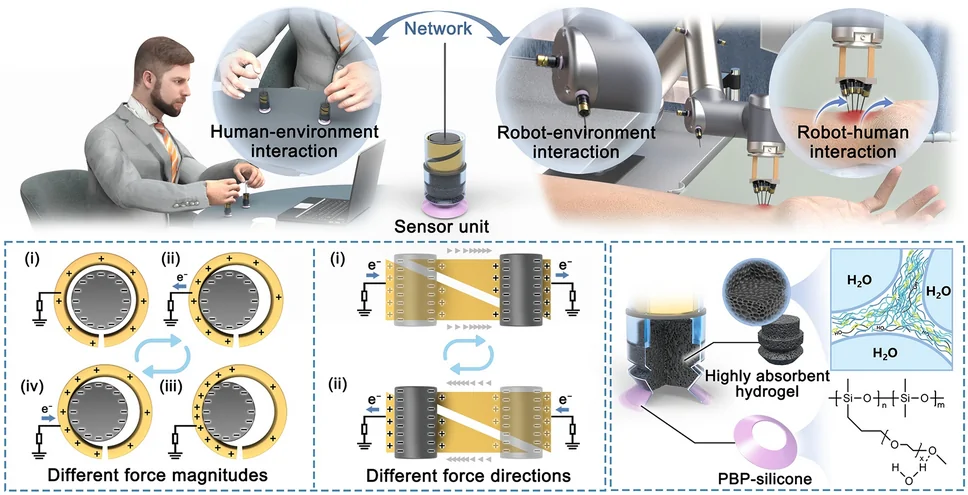

## Introduction

In recent years, the growing integration of robotics and intelligent systems into daily life has led to an increasing number of interaction scenarios involving humans, machines, and complex environments [1]. From collaborative robots in industrial settings to wearable devices and remote medical tools, seamless and efficient interaction has become a defining feature of next-generation intelligent systems [2, 3]. This evolution highlights a pressing need for reliable tactile sensing interfaces that can serve as a bridge between physical contact and digital perception [4–7]. Among various solutions, the concept of a versatile, high-precision tactile interface—capable of adapting to diverse surfaces with adjustable configurations while providing high-resolution, omnidirectional force and motion sensing—has attracted significant attention [8]. To address this, an ideal tactile interface should feature a series of universal sensor units that are not only flexibly and reversibly deployable across varied [9], often irregular or even dynamic surfaces, but also capable of accurately sensing omnidirectional forces and motion. However, achieving both of these points remains significant challenge for current sensing technologies.

Addressing the first challenge, traditional flexible sensor arrays often lack unit-level mobility, limiting their versatility to complex surfaces or tasks [10, 11]. In contrast, whisker-inspired configurations feature compact, independently deployable units well suited for constructing reconfigurable tactile interfaces with diverse spatial distributions and functionalities. However, effective integration requires more than structural flexibility—it demands reversible, stable anchoring across diverse surfaces while preserving compactness [12, 13]. Existing strategies—such as microspine [14], dry adhesive [15], electrostatic pad [16], conformal grip actuator [17], and vacuum suction cups [18]—often compromise between aspects such as actuating source independence, portability, structural simplicity, anchor strength, surface adaptability, and manufacturing simplicity (Table S1), making them difficult to meet the sensor units’ demands [19]. Liquid-sealed suction cups [20], while offering better anchoring strength and surface adaptability than vacuum ones, typically rely on external water supply systems, which also hinders compact integration. Therefore, how to better balance the performance across all aspects remains a significant challenge.

The second challenge involves achieving precise omnidirectional force and motion sensing. Conventional 2D sensors are limited to single-axis force detection [21–23], while many 3D designs lack the ability to resolve motion trajectories, limiting their utility in dynamic interactions [24, 25]. Whisker-inspired sensors overcome some of these constraints by coupling multidirectional forces and motions with the wide range of deformations of their high-compliance whiskers [26]. However, current whisker sensor implementations still face trade-offs: Clip-type designs are often limited in directionality [27]. Although increasing the electrode count can improve spatial resolution, it comes at the cost of signal complexity and system miniaturization [28]. As a result, achieving compact, high-resolution omnidirectional sensing remains a key bottleneck (Table S2). Moreover, to further enhance sensing sensitivity and output, recent studies have emphasized the incorporation of high-electronegativity materials—such as fluorides [29], oxides [30], and emerging 2D materials [31]—into dielectric layers to improve polarization and charge trapping efficiency. Among these, MXenes (*M*_*n*+1_*X*_*n*_*T*_*x*_) stand out due to their high conductivity, mechanical robustness, abundant surface terminations, and strong electronegativity, which collectively contribute to significant improvements in sensor performance [32, 33]. Integrating MXene-based materials into dielectric structures is thus a key strategy for developing compact and high-resolution sensing devices [34, 35].

In this study, we present a reconfigurable omnidirectional triboelectric whisker sensor array (RO-TWSA) consisting of multiple independent sensing units that collectively overcome the aforementioned-challenges. First, an untethered hydro-sealing vacuum sucker (UHSVS) deploys a highly absorbent cylindrical foamy hydrogel and a hydrophilic annular poly(dimethylsiloxane-*b*-ethylene oxide) (PBP)-silicone inside and along the lower edge of the suction cup, respectively, enabling efficient water storage, controllable extrusion, sustained retention and rapid recovery. Through such liquid-mediated vacuum adsorption, the UHSVS enables sensor units to achieve strong and reversible adsorption to multiple surfaces, while preserving their anchoring force after 200 adsorption cycles. Second, a dual-triangle triboelectric whisker structure (TWS) embedded with MXene/silicone nanocomposite allows a high-precision sensing of the magnitude and direction of omnidirectional force and motion simultaneously by only two electrodes, achieving an impressive force threshold of 0.024 N and an angular resolution of 5°. Composed of such a series of sensor units, the RO-TWSA can be flexibly reconfigured as needed on a variety of complex surfaces with interaction requirements as a versatile sensing interface to record dynamic interaction forces and motion information. Therefore, our RO-TWSA is applicable in scenarios such as easily deployed teleoperation (human–environment interaction), resolution-adjustable robotic arm palpation (human–robot interaction), and function-switchable robotic autonomous environmental exploration (robot–environment interaction), respectively. These unprecedented performance and versatility in real-world interaction scenarios hold great potential for future applications in human–machine–environment interaction perception.

## Results and Discussion

### Structure and Working Mechanism of the RO-TWSA

**Fig. 1:**
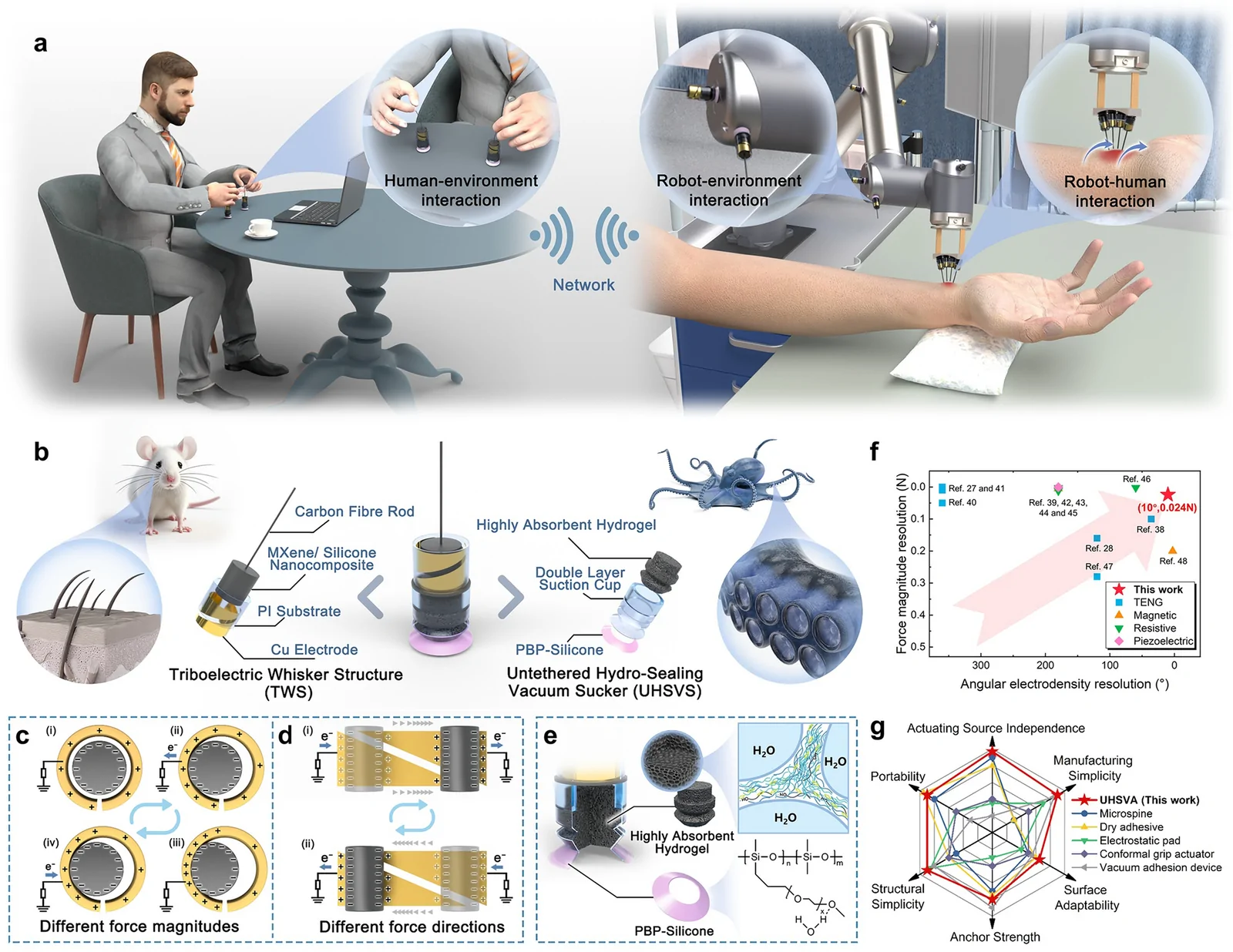
Concept, structure, and mechanism of the RO-TWSA. **a** Application of the RO-TWSA in remote robotic arm manipulation for tactile diagnostics. **b** Structure schematic of the sensor unit and corresponding bionic prototypes. Working principle of the TWS under different **c** force magnitudes and **d** directions. **e** Chemical structures of highly absorbent hydrogel and PBP-silicone. **f** Force magnitude and angular resolution of the sensor unit compared to previously reported whisker sensors. **g** Radar chart comparing the performance metrics of the UHSVS with other anchoring methods

As shown in Fig. 1, the RO-TWSA consists of a series of sensing units that integrate a triboelectric whisker structure with an untethered hydro-sealing vacuum sucker. This bioinspired configuration recapitulates the mechanosensory function of rat whiskers and the reversible adsorption capability of octopus suckers, aiming to create a sensor unit that combines rich tactile perception with versatile deployment. The RO-TWSA can be utilized in various interaction scenarios between human–robot–environment. A typical application involves a doctor using a portable RO-TWSA device to remotely control a robotic arm equipped with a RO-TWSA-based palpation end-effector (Fig. 1a), while the robotic arm body is also distributed with RO-TWSA for detecting and preventing accidental collisions.

The triboelectric whisker structure mimics rat vibrissae [28], comprising a carbon fiber rod embedded in a cylindrical MXene/silicone nanocomposite dielectric layer (Fig. 1b and Note S1). A polyimide ring with two mirror-symmetric triangular copper electrodes surrounds this structure (Fig. S1). Operating in single-electrode mode (Fig. 1c, d), the configuration generates dual outputs: The sum of signals reflects the contact force magnitude, while their difference indicates deflection direction, enabling precise omnidirectional sensing.

As shown in Fig. 1c, when an external force deflects the carbon fiber whisker, the cylindrical MXene/silicone nanocomposite layer beneath approaches the copper electrodes. Owing to the much higher electronegativity of the MXene/silicone nanocomposite, free electrons on the copper surface transfer to its lowest unoccupied molecular orbital [36, 37], resulting in a negative charge on the nanocomposite and a positive charge on the copper (Fig. 1c(i)). As the force increases, the decreasing distance between the silicone and the electrode enhances charge transfer (Fig. 1c(i)–(ii)). Continued deflection causes the silicone to contact and then compress against the electrode, enlarging the contact area and further increasing charge transfer and output voltage (Fig. 1c(ii)–(iii)). Maximum charge transfer is achieved upon full contact. When the force is released, the elastic restoring force of the carbon fiber rod and silicone layer drives the nanocomposite to detach from the electrode, reversing the charge flow (Fig. 1c(iv)). The diagonally mirror-symmetric triangular electrodes create a lateral-deflection–sensitive structure that, upon whisker bending, produces direction-specific voltage differences via spatially asymmetric contact areas, enabling directional identification through differential signal analysis (Fig. 1d).

To benchmark its competitive precision and functionality in complex interaction scenarios, Fig. 1f compares the force sensitivity and angular resolution of sensor unit with other whisker sensors. Despite using only two electrodes, the sensor unit achieves a high angular resolution of 5° and an impressive force threshold of 0.024 N, underscoring its advantages in precise, real-time sensing for complex human–robot–environment interactions [27, 28, 38–48].

Inspired by the adsorption mechanism of octopus suckers, the lower suction cup replicates the formation of a sealed cavity and mucus secretion to enhance attachment [49]. As shown in Fig. 1e, the UHSVS consists of a dual-layer suction cup, a highly absorbent hydrogel, and a hydrophilic PBP-silicone layer. The triboelectric whisker structure is securely bonded to the UHSVS via silicone adhesive (Sil-Poxy). As part of the bottom pad of the suction cup, the hydrophilic silicone, synthesized by incorporating PBP into conventional silicone, improves water affinity and enhances adsorption performance [50].

The suction cup’s effectiveness relies on the interplay between the hydrogel and hydrophilic silicone. Upon compression, the hydrogel releases water to form a stable liquid seal, while the PBP-silicone ring retains moisture, reducing water loss and ensuring repeatable performance over multiple cycles. Detachment is achieved by pressing the dual-layer folds to admit air and break the seal. The hydrogel then rapidly reabsorbs water and restores its shape, enabling reuse. This integrated mechanism of water expulsion, retention, and recovery provides a robust, reversible sealing strategy, supporting efficient and repeatable anchoring without external water input. As illustrated in Fig. 1g, the UHSVS demonstrates superior performance in terms of actuator independence, manufacturability, surface adaptability, anchoring strength, structural simplicity, and portability, making it well suited for deployment in diverse environments.

### Output Performance and Characteristics of the TWS

**Fig. 2:**
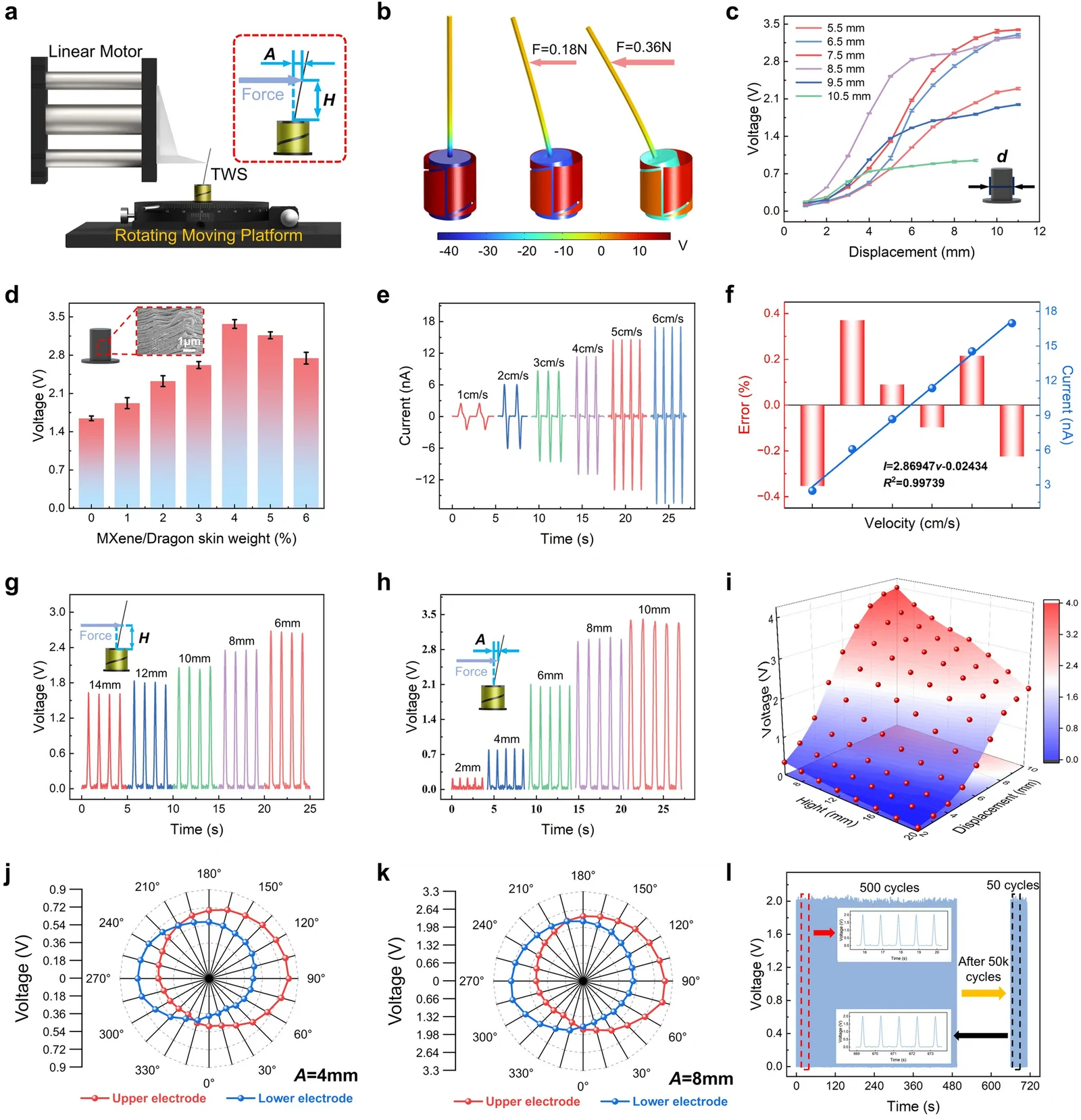
Performance of the TWS under different load parameters. **a** Schematic diagram of the experimental setup and deformation state under external load. **b** COMSOL simulation of potential distribution between the electrodes and silicone under different force magnitudes. **c** Voltage response of the TWS with varying silicone diameters (5.5, 6.5, 7.5, 8.5, 9.5, and 10.5 mm) at *H* = 10 mm and *v* = 3 cm s^−1^. **d** Open-circuit voltage of the TWS under different MXene nanosheets doping concentrations. **e** Current response to varying contact velocity from 1 to 6 cm s^−1^. **f** Linear fitting of short-circuit current as a function of contact velocity. **g** Voltage response to different contact heights from 14 to 6 mm. **h** Voltage response to varying load displacement from 2 to 10 mm. **i** Relationship between open-circuit voltage, load displacement and contact height. Open-circuit voltage responding to stimuli at different angles under the conditions of **j** *A* = 4 mm, **k** *A* = 8 mm. **l** Durability of the TWS tested for 50,000 cycles

To validate its applicability in real-world interactive scenarios, the electromechanical performance of the sensor unit is comprehensively characterized, including sensitivity, resolution, directional response, environmental adaptability, and long-term stability. The TWS is mounted on a rotating platform and driven by a linear motor, while its electrical output is measured using a high-resistance electrometer and visualized in real time via LabVIEW (Fig. S2). Figure 2a illustrates the experimental setup, where *H* represents the vertical distance from the carbon fiber contact point to the MXene/silicone layer, and *A* denotes the displacement at the force application point.

Given its slender geometry and small deformations, the TWS follows the Euler–Bernoulli beam model (Note S2), and COMSOL simulations were conducted to analyze the displacement profiles along the whisker under varying forces (Fig. S3). As both electrodes are identically designed, performance analysis focuses on one. The working mechanism is schematically illustrated in Fig. 1c, d, and further validated by the COMSOL-simulated potential distribution between the electrodes and the silicone, as shown in Figs. 2b and S4. The details of COMSOL simulation for the potential distribution are shown in Note S3.

To optimize sensitivity, the influence of structural parameters on voltage output is examined. As shown in Fig. 2c, the open-circuit voltage first increases and then decreases with increasing diameter of the MXene/silicone nanocomposite at *H* = 10 mm and *v* = 3 cm s^−1^. The voltage peaks at a diameter of 7.5 mm, where the trade-off between contact area and separation efficiency yields optimal output (Fig. S5). With this configuration, the TWS can detect forces as low as 0.024 N, demonstrating high sensitivity and resolution (Fig. S6, Movie S1).

In terms of material composition, a MXene additives concentration of 4% proves optimal at *H* = 10 mm, *A* = 10 mm, and *v* = 3 cm s^−1^. As shown in Fig. 2d, the open-circuit voltage increases from 1.64 to 3.37 V as the MXene content rises from 0 to 4 wt%, representing a 2.05-fold enhancement compared to pure silicone rubber. However, further increasing the concentration beyond 4 wt% (e.g., 5% and 6%) leads to a decline in electrical output, likely due to the aggregation of excessive nanosheets. This aggregation may reduce ion mobility and hinder charge migration, thereby decreasing the overall performance of the TWS [51]. Additionally, to confirm the binding of MXene and gain insights into the surface chemistry, we have conducted X-ray photoelectron spectroscopy (XPS) analysis. The XPS spectrum clearly reveals the presence of Ti, C, O, and Si elements, confirming the successful incorporation and surface binding of MXene nanosheets within the silicone matrix (Fig. S7).

The short-circuit current of the TWS is measured at different contact velocities with *H* = 10 mm and *A* = 7 mm. As shown in Fig. 2e, the current increased from 2.5 to 17.1 nA as the velocity rose from 1 to 6 cm s^−1^. A leave-one-out cross-validation (LOOCV) approach is used to analyze this relationship, yielding a linear fit of *I* = 2.86947*v* − 0.02434 with a correlation coefficient *R*^2^ = 0.99739 (Fig. 2f). The relative error is below 0.09% at 3 cm s^−1^ and 0.37% at 2 cm s^−1^, likely due to high-frequency motor vibrations [52]. Meanwhile, the open-circuit voltage remains stable around 2.6 V across different velocities (Fig. S8), indicating speed-independent voltage output.

To assess the effect of contact position and force magnitude, voltage responses under varying contact heights and displacements are analyzed. As the contact height decreases (with fixed *A* = 6 mm), increased contact area enhances charge transfer and voltage output (Fig. 2g). At *v* = 3 cm s^−1^ and *H* = 10 mm, the voltage increases from 0.23 to 3.36 V as displacement rose from 2 to 10 mm (Fig. 2h). Combined effects of displacement and contact height confirm a consistent trend of increasing voltage with larger displacement and lower contact point (Fig. 2i).

Figure 2j, k shows the voltage signals of two electrodes under displacements of 4 and 8 mm in different directions (with 15° intervals and *H* = 10 mm). Taking the seam line as the 0° reference point, the angle increases in a counterclockwise direction when viewed from above. Under different load directions, the signal output gradually increases with the increase in the contact area corresponding to the sensing electrode. The test results for displacements of 2, 6, and 10 mm are shown in Fig. S9. The TWS can accurately detect both the magnitude and direction of external loads. (Detailed explanation is shown in Note S4.) Moreover, by further subdividing the experimental directional intervals, the TWS achieves an angular resolution of 5° (Fig. S10 and Movie S1).

Environmental adaptability is tested under controlled humidity. The open-circuit voltage decreases as relative humidity increases from 55 to 95% due to surface charge dissipation from water layer formation [53], yet maintains 1.51 V at 95%, demonstrating robust performance under high humidity (Fig. S11). Additionally, durability is verified by 50,000 cycles at *H* = 10 mm and *A* = 6 mm (Fig. 2l). The TWS maintains stable output (~ 2.05 V), confirming strong durability and long-term stability of both the device and MXene material.

### Fabrication and Characterization of the UHSVS

**Fig. 3:**
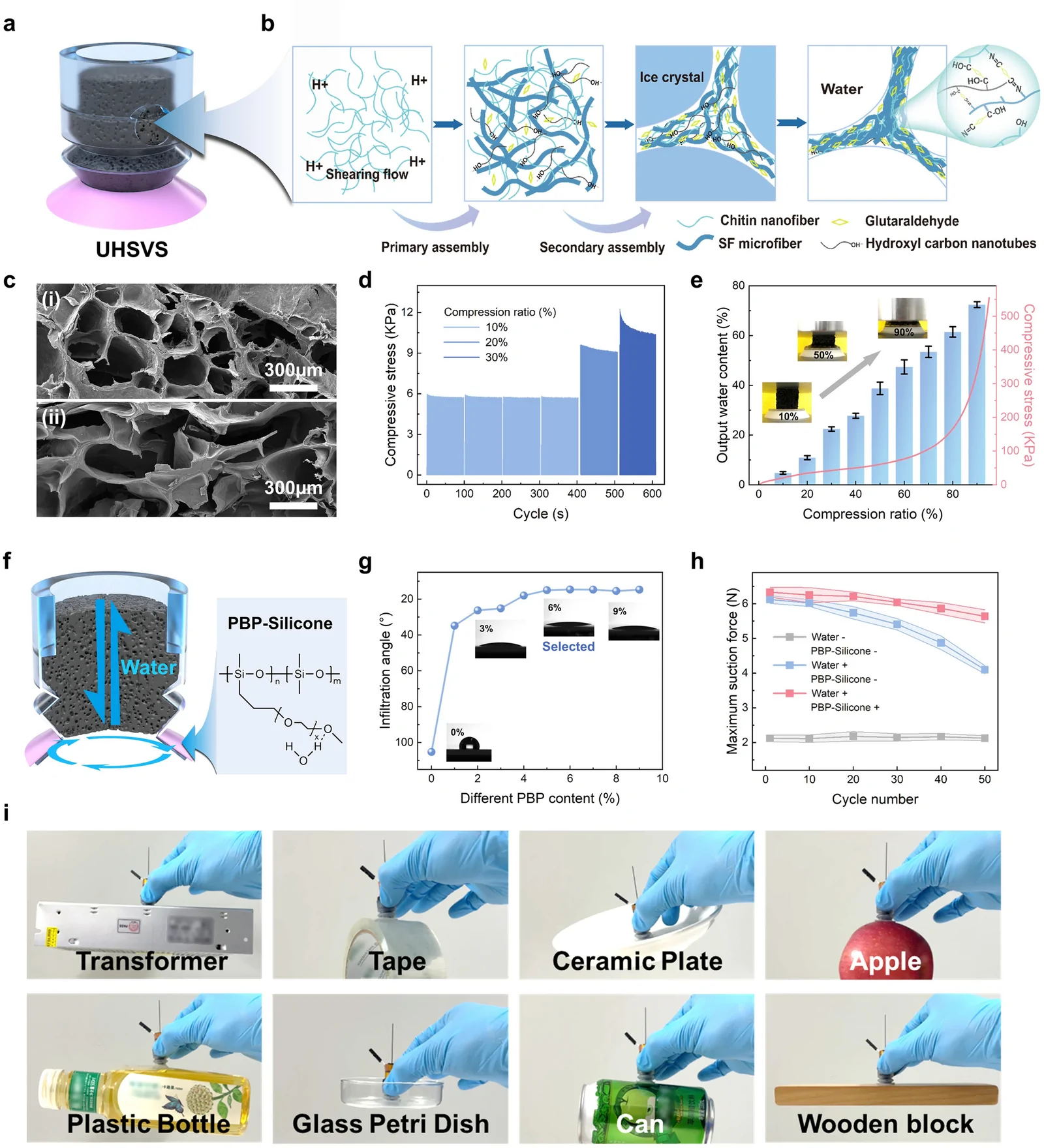
Design and characterization of the UHSVS. **a** Structural design of the UHSVS. **b** Schematic illustration and chemical structures of the highly absorbent hydrogel. **c** Skeleton structures of highly absorbent hydrogel observed by SEM (scale bars, 300 nm). (i) Before compression and (ii) at 50% compression. **d** Cyclic compressive stress–strain curves of highly absorbent hydrogel at different strains. **e** Water output capacity of the highly absorbent hydrogel at different pressures. Each bar represents the mean ± standard deviation (*n* = 5). Insets depict the corresponding compression states. **f** Structural illustration of the water-sealing mechanism in the UHSVS. (Left) Schematic of internal water flow dynamics enabled by the hydrogel and suction cavity. (Right) Molecular structure of PBP-silicone. **g** Contact angle test on PBP-silicone surface with different PBP concentrations (from 0 to 9%, with 1% interval). **h** Suction force versus number of suction cycles (0–50) for three suction cup configurations (hydrogel without water and without PBP-silicone, hydrogel with water and without PBP-silicone, and hydrogel with water and with PBP-silicone). **i** Demonstration of UHSVS adsorption on diverse surfaces, including a plastic bottle, tape, glass petri dish, ceramic plate, can, apple and wooden block

Inspired by the adaptive adhesion of natural octopus, the sensor units of RO-TWSA incorporate a biomimetic design featuring a dual-layer suction cup. To support its robust and reversible deployment on different surfaces, we introduce a highly absorbent hydrogel and a PBP-silicone ring, achieving reliable and reversible liquid storage and sealing under deformation and repeated use through material design (Fig. 3a). Specifically, the hydrogel needs to break through the inherent trade-off between water retention and structural stability. To overcome this challenge, we integrate chitin, silk fibroin, and hydroxylated carbon nanotubes (CNTs) to synergistically optimize the absorption capacity, elasticity, and structural integrity of the hydrogel under cyclic deformation. The fabrication process is illustrated in Fig. 3b. Chitin solution was mixed with acetic acid to adjust pH, followed by the addition of silk fibroin (SF), hydroxylated carbon nanotubes, and glutaraldehyde. After vigorous stirring and centrifugation, the mixture was molded, frozen at − 20 °C for 8 h, thawed in ethanol, and soaked in deionized water (Fig. S12).

The resulting hydrogel demonstrates a porous yet tough structure that maintains its performance under repeated mechanical loading. Scanning electron microscopy (SEM) images reveal that the highly absorbent hydrogel, both before compression and at 50% compression, exhibits a highly interconnected porous polymeric network with large pore sizes and high porosity (Fig. 3c). Quantitative analysis of pore size distribution is conducted using ImageJ software based on representative SEM images prepared with and without glutaraldehyde cross-linking (Fig. S13), yielding a porosity of around 76.2% comparable with macroporous hydrogel systems [54]. This is mainly due to the semirigid nature of chitosan chains and their extensive hydrogen bonding, which exert minimal inhibition on ice crystal growth and thus result in relatively large ice crystals [55–57]. (Detailed explanation is shown in Note S5.) Fourier-transform infrared spectroscopy (FTIR) analysis (Fig. S14) reveals the presence of an O–H stretching vibration peak near 3500 cm^−1^ associated with unbound O–H groups within the gel matrix. The broad absorption band around 3400 cm^−1^ corresponds to overlapping O–H and N–H stretching vibrations, while the shoulder near 3300 cm^−1^ is primarily attributed to free O–H stretching. The addition of glutaraldehyde effectively reduced the concentration of free hydroxyl groups and facilitated the establishment of a more compact hydrogen bonding network. The hydrogel exhibits exceptional resilience under cyclic loading exceeding 90%, as demonstrated in Fig. 3d, e and Movie S2. Furthermore, the rapid hydro-swelling of the hydrogel is crucial for maintaining its high compression resistance, which allows the hydrogel’s rapid water expulsion under compression and swift recovery upon rehydration, facilitating reversible liquid sealing during anchoring cycles (Fig. S15 and Movie S3). A hydrogel with superhydrophilicity is generated, which arises from the synergistic effect of the intrinsically hydrophilic chitin nanofibers and the highly porous network structure [58, 59]. This enables rapid water absorption and enhanced interfacial interactions (Fig. S16). Water retention experiments are conducted by placing the hydrogel inside the double suction cup for 7 days (Fig. S17). The hydrogel retains over 83% of its initial water content, confirming its stability and ensuring consistent suction performance over time, which enhances the long-term durability and reliability of the sensor array in dynamic environments.

Collaboratively, the PBP-silicone ring can help to achieve an effective liquid seal, as shown in Fig. 3f. The hydrophobicity of the silicone surface presents a significant challenge to achieving effective liquid sealing, thus reducing suction force [50]. To overcome this challenge, the silicone is chemically modified by incorporating a hydrophilic copolymer, poly(dimethylsiloxane-b-ethylene oxide) (PBP) [60], which enhances hydrophilicity and facilitates the formation of a more stable liquid seal. (Detailed explanation is shown in Note S5.) Fig. 3g shows that as the PBP content increases, the hydrophilicity of the silicone surface improves significantly. However, when the PBP concentration exceeds 6%, no further significant reduction in contact angle is observed. In addition, higher PBP concentrations lead to a gradual increase in viscosity, which impairs the composite’s castability and results in inhomogeneous films. Therefore, a 6% PBP concentration is selected as the optimal value for enhancing silicone hydrophilicity, striking an ideal balance between hydrophilicity, processability, and material efficiency.

To evaluate suction force, we refer to the methodologies [12, 13] and construct the experimental setup as shown in Fig. S18. The suction cup is pressed at a controlled rate of 10 mm/min until the applied force reached 6 N, after which it is lifted at the same rate. During detachment, force variation is continuously monitored and recorded to capture the suction force dynamics. This procedure is further demonstrated in Movie S4. Furthermore, we conduct quantitative comparisons on different material surfaces, and the force–displacement curves for these tests are provided in Fig. S19. The results confirm that the UHSVS exhibits superior absorption performance across multiple tested surfaces, demonstrating both its material adaptability and strong suction performance.

The maximum adsorption force is measured during this process for three different suction cup configurations: (1) a suction cup with water-free, highly absorbent hydrogel and non-hydrophilic silicone ring; (2) a suction cup with highly absorbent hydrogel containing water and non-hydrophilic silicone ring; and (3) a suction cup with highly absorbent hydrogel containing water and hydrophilic silicone ring. The maximum suction forces recorded by the force sensor for each configuration are shown in Fig. 3h. The incorporation of the highly absorbent hydrogel and the PBP-silicone ring facilitates the formation of a robust liquid seal at the interface, greatly enhancing the adsorption force. Additionally, the robust liquid seal also facilitates the water to be fully recycled and reused by the hydrogel, which mitigates the decline in adsorption force over multiple anchoring cycles. To evaluate the effect of water content, suction tests are conducted using hydrogels with varying moisture levels. As shown in Fig. S20, suction force increases with higher water content, underscoring the critical role of hydration in maintaining stable performance. Over 200 suction cycles, the anchoring force remains stable at approximately 4.6 N (Fig. S21).

Additional experiments are conducted to assess the effect of pull-off speed. As shown in Fig. S22, the suction force–displacement curves are recorded at pull-off speeds of 10, 20, 30, 50, and 100 mm min^−1^, revealing a slight decrease in suction force with increasing speed. The influence of suction cup diameter is also examined. As shown in Fig. S23, larger diameters correspond to higher maximum suction forces. However, considering practical constraints such as device compactness and integration, a 15 mm diameter was selected as the optimal size for sensor fabrication.

Further validation of the UHSVS’s high adaptability and high adsorption force-to-weight ratio is conducted in real-world scenarios. The UHSVS achieves reliable anchoring through passive negative pressure and a peripheral liquid seal formed by expelled interfacial water during compression. While the detachment is realized by slightly pressing the lower fold of the double suction cup, allowing air inflow to neutralize the pressure difference. This process is fast, tool-free, and fully reversible. After detachment, the soft structure recovers quickly, and the hydrogel reabsorbs the water, enabling repeatable use. As shown in Fig. 3i and Movie S5, the sucker adsorbs to various materials. Despite weighing only 2.4 g, it can lift up to 560 g—approximately 230 times its own weight—demonstrating both its exceptional adsorption performance and compact design.

### Easily Deployed Teleoperation Enabled by RO-TWSA

The RO-TWSA holds great potential in human–environment interaction applications. With a compact profile and reconfigurable omnidirectional sensing capability, the RO-TWSA enables seamless integration onto diverse daily objects, allowing users to create portable teleoperation interfaces in diverse environments on demand. Given the relatively poor portability of existing joysticks and teleoperation platforms, RO-TWSA could be a promising solution to fill this gap.

**Fig. 4:**
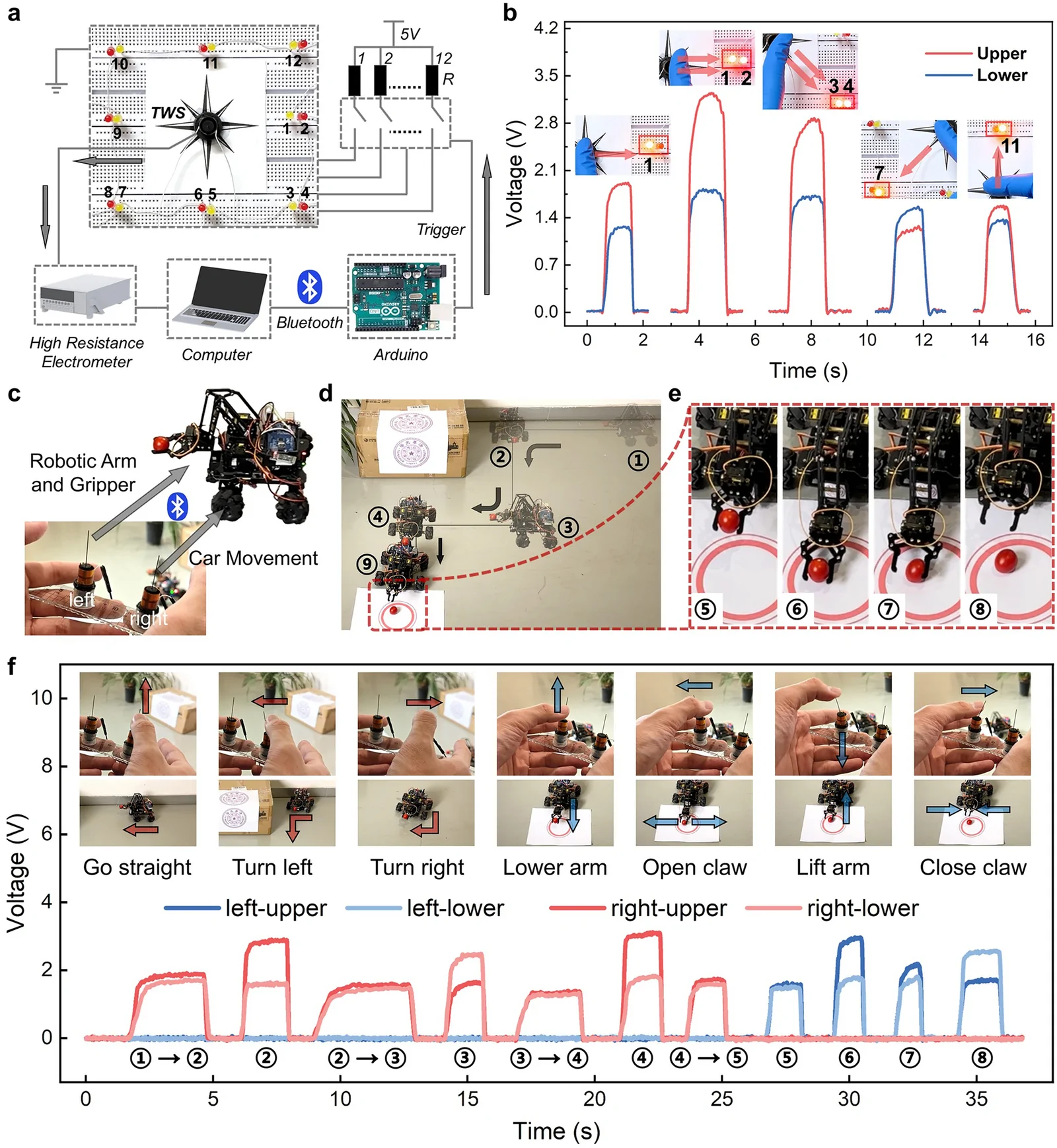
Easily deployed teleoperation enabled by RO-TWSA. **a** Electronic module used for controlling LED lights. **b** Demonstration of the TWS as a load switch controller and its corresponding output voltage signal. **c** Demonstration of using RO-TWSA to control a car equipped with a robotic arm. **d** Photographic sequence showing the motion trajectory of the controlled car. **e** Images capturing the robotic arm and claw in motion. **f** Real-time signals of the RO-TWSA corresponding to the control process (the inset shows the state of the car and operator’s hand)

To demonstrate the feasibility of RO-TWSA as an interactive interface for easily deployable teleoperation, we first evaluate the interaction capability of a sensing unit experimentally using light-emitting diodes (LEDs) controlled by TWS signals. LEDs are arranged around the TWS at intervals of 45° (Fig. 4a). During the experiment, real-time voltage signals from the TWS are acquired by the electrometer. These signals are processed and visualized through a MATLAB interface embedded in LabVIEW. Control commands are transmitted from MATLAB to an Arduino Uno R3 via Bluetooth to control the illumination of the corresponding LEDs. Each direction is assigned two LEDs, and the number of LEDs illuminated correlates with the peak voltage magnitude of the TWS. A sliding window algorithm is employed for signal sampling to reduce interference from transient fluctuations and noise.

As shown in Fig. 4b, when the user deflects the whisker rod in eight different directions with varying force magnitudes, distinct voltage pulses are generated, triggering the corresponding LEDs (Movie S6). This real-time mapping of tactile stimuli to visual output not only confirms the sensor unit’s directional sensitivity and resolution but also establishes a closed-loop feedback pathway essential for dynamic robotic control.

To further demonstrate the role of RO-TWSA as a compact and portable teleoperation interface for human–environment interaction, we develop a flexible reconfigurable multi-channel control system based on RO-TWSA. This system enables users to send tactile signals to remote robotic devices in real time. As illustrated in Fig. 4c, electrical signals are collected via an electrometer and processed in MATLAB. The resulting control commands are wirelessly transmitted via Bluetooth to an Arduino Uno R3, which governs the robot’s actuators to perform corresponding tasks. We mount two sensor units onto a daily object—a plastic ruler—transforming it into an interactive teleoperation handle. This plug-and-play strategy allows for rapid assembly of control interfaces without structural redesign or costly customization, enabling low-barrier, reconfigurable teleoperation across diverse real-world environments.

Using this flexible interface, we demonstrate that a user can control a robotic car and its gripper by manipulating two sensor units (Fig. 4d). Specifically, the right-hand sensor unit is used to control the movement of the car, while the left-hand sensor unit governs the robotic arm and gripper actions. For precise control, four angular positions on the left sensor unit (0°, 90°, 180°, and 270°) correspond to extending the robotic arm, opening the gripper, retracting the arm, and closing the gripper, respectively (Fig. 4e). Likewise, the same four directional positions on the right sensor unit are mapped to forward movement, left turn, backward movement, and right turn of the car. Figure 4f presents the signal outputs from two sensor units, along with the associated finger gestures and corresponding movements. Real-time demonstrations of car and gripper control are provided in Movie S7. Moreover, the RO-TWSA can anchor to various surfaces, highlighting its practical potential for active, surface-mounted control in versatile human–environment interactions.

### Resolution-Adjustable Robotic Arm Palpation Enabled by RO-TWSA

The RO-TWSA can bring attractive possibilities to human–robot interaction applications. The reconfigurable architecture of the RO-TWSA consists of a series of reversibly deployable sensor units, allowing it to flexibly adjust diverse array parameters such as spatial resolution, coverage area, and contact geometry. Therefore, the RO-TWSA can flexibly adapt to various human–robot interaction scenarios and tasks without requiring hardware redesign, which is particularly beneficial for tasks requiring variable perceptual granularity.

A representative application is robotic palpation, where biological tissues often exhibit heterogeneous stiffness, shape, and spatial scale [61]. Effective detection demands that the sensor array first detect the approximate location of the abnormal part (requires a high sensing space range) and then determine the specific contour or stiffness of the abnormal part through a high-resolution local scan (requires a high sensing resolution). The RO-TWSA has the potential to fulfill this requirement with a compact and simple structure by enabling rapid configuration adjustments.

**Fig. 5:**
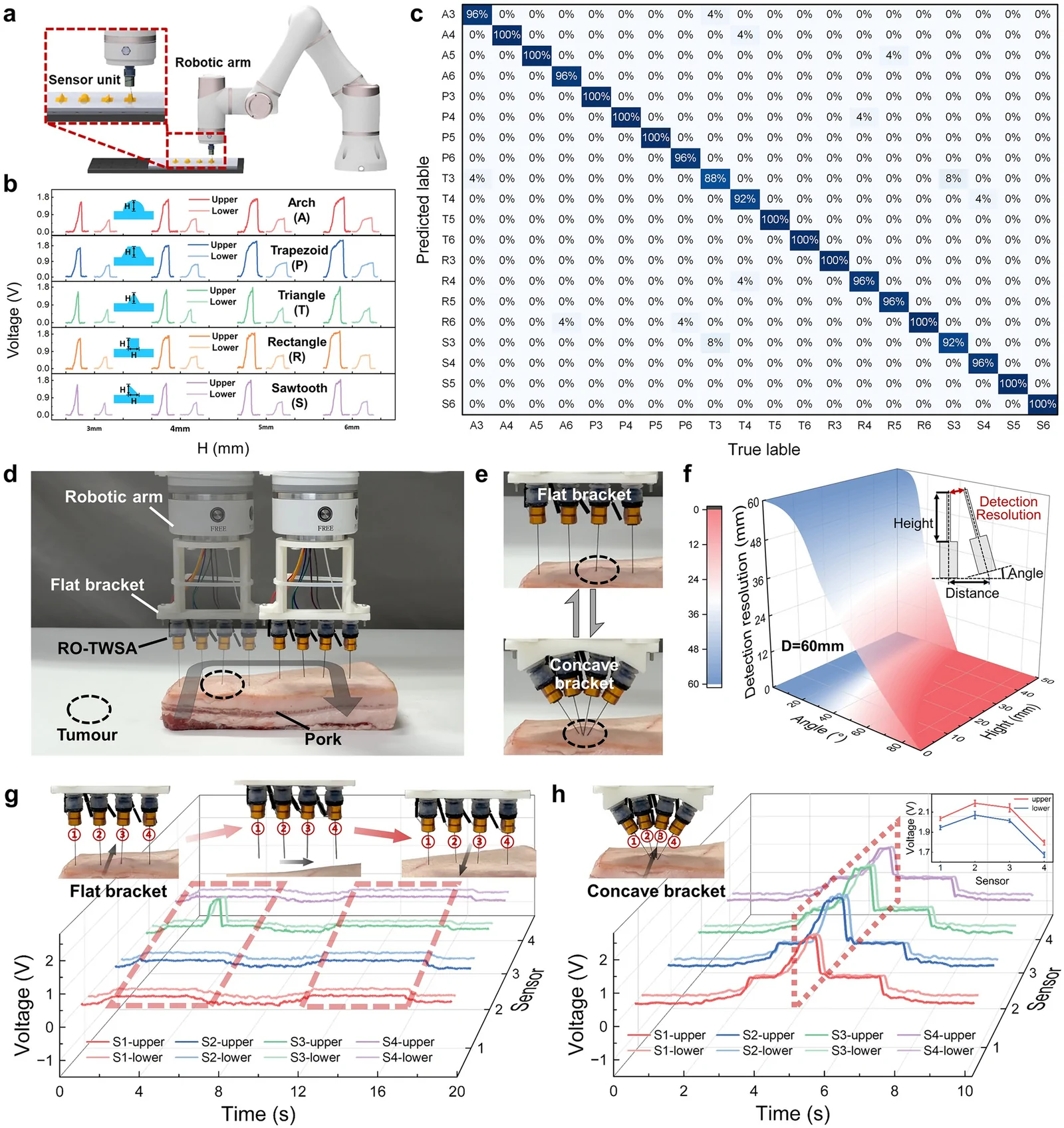
Resolution-adjustable robotic arm palpation enabled by RO-TWSA. **a** Experiment setup for surface topography classification. **b** Output of the sensor unit during sliding over twenty samples with different textures. **c** Confusion matrix of texture classification. **d** Experimental setup of tactile diagnostic operations by a robotic arm. **e** Different brackets for palpation tasks. **f** Theoretical analysis of the relationship between height, distance, angle, and detection resolution in the placement of two sensor units. Real-time monitoring of the voltage output of the RO-TWSA mounted on **g** the flat bracket (insets depict the corresponding tactile diagnostic operations) and **h** the concave bracket (inset depicts maximum voltage during palpation, and each bar represents the mean ± standard deviation (*n* = 5))

In clinical or biomedical contexts, tactile interfaces must distinguish subtle variations in subcutaneous structures, which vary in shape, size, and mechanical properties. To evaluate the tactile performance of RO-TWSA in robotic palpation, we first verify a sensor unit’s ability to recognize fine surface textures—a critical prerequisite for robotic palpation. A controlled testing platform is constructed in which the sensor unit slides across five types of 3D-printed textured modules—arch (A), trapezoid (P), triangle (T), rectangle (R), and sawtooth (S)—each available in five height variants (*H* = 3, 4, 5, and 6 mm). The carbon fiber whisker tip is aligned to the start point of each texture to ensure continuous contact throughout movement (Fig. 5a). As shown in Fig. 5b, the two electrodes generate distinguishable voltage signals for each texture profile and scale. These temporal signal patterns serve as inputs to a convolutional neural network (CNN) for texture classification (Fig. S24 and Note S7), achieving an overall accuracy of 97.4% (Fig. 5c). This high classification accuracy not only validates the sensor unit’s sensitivity to fine texture variations but also underscores its scalability for array-level tasks. Specifically, the RO-TWSA system can enable resolution-adjustable high-sensitive tactile sensing by leveraging its reversibly deployable sensor units.

To further validate the tactile performance of RO-TWSA in realistic medical scenarios, we simulate a two-stage robotic palpation task commonly encountered in clinical diagnostics: The first stage aims to broadly localize potential subcutaneous anomalies across a wide area, while the second focuses on delineating their contours with high sensing resolution. As shown in Fig. 5d, we construct a robotic diagnostic platform using porcine epidermal tissue, which closely mimics the mechanical properties of human skin [62]. A 3D-printed hemispherical part is embedded beneath the tissue to simulate the tumor. Four sensor units are mounted on a set of brackets with different shape parameters to form a palpation head mounted at the end of the robotic arm. The palpation head can be adapted to different diagnostic stages by replacing the different brackets and redeploying the sensing units (Fig. 5e). A theoretical analysis of how bracket parameters affect spatial resolution is illustrated in Figs. 5f and S25.

In the first phase of palpation, the robot employs a flat bracket to scan the tissue at a constant speed (Movie S8). When surface irregularities are encountered, electrical signal peaks are generated, indicating the presence of anomalies (Fig. 5g). To improve spatial resolution in the second phase, we replace the flat bracket with a concave one (Fig. 5h), thereby enabling more accurate delineation of the tumor morphology. These results demonstrate RO-TWSA’s applicability in sequential diagnostic stages and thus in other human–robot interaction scenarios, through hardware reconfiguration without structural redesign.

### Function-Switchable Robotic Autonomous Environmental Exploration Enabled by RO-TWSA

The RO-TWSA can also serve as a versatile sensing interface for robot–environment interaction applications. To support robot autonomous environmental exploration in complex scenarios, the RO-TWSA can be integrated into the robot platform, enabling the system to quickly switch functions in response to different task requirements, such as detecting obstacles or mapping environments. To validate this capability, four sensor units are integrated into a mobile robot, demonstrating how the RO-TWSA enables the robot to flexibly switch between different functions, thereby facilitating multifunctional robot–environment interactions based on simple structure.

**Fig. 6:**
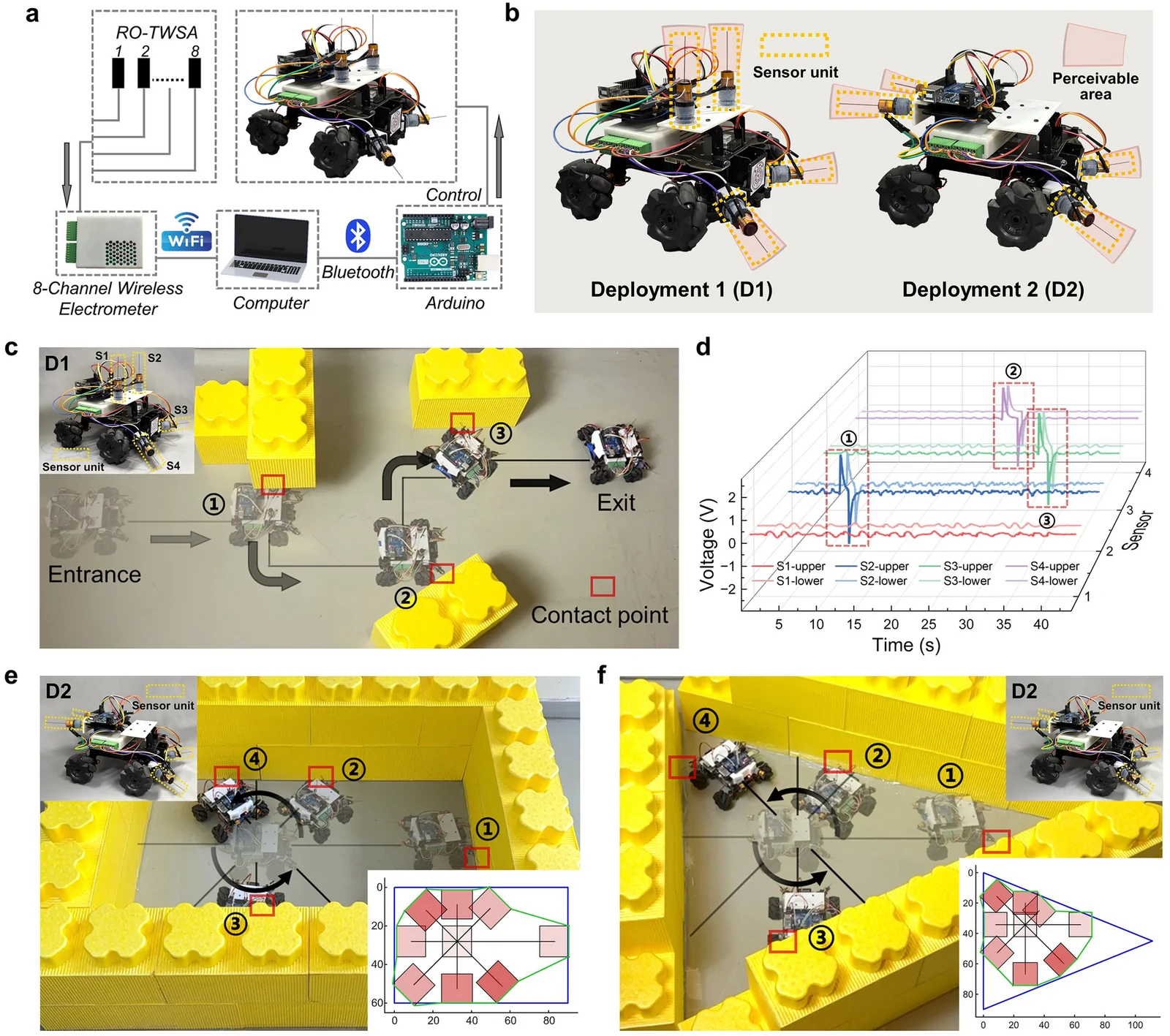
Function-switchable robotic autonomous environmental exploration enabled by RO-TWSA. **a** Schematic diagram of the mobile robot equipped with the RO-TWSA, comprising 4 sensor units and a wireless electrometer board. **b** Photographs of 2 types of RO-TWSA deployment. **c** Motion trajectory during autonomous obstacle avoidance in D1 deployment. **d** Real-time voltage signals acquired from the electrometer board in the process of autonomous obstacle avoidance. Motion trajectories for map construction in D2 deployment and corresponding simulation results for a **e** rectangle area and a **f** triangle area

The RO-TWSA perception system consists of four sensor units installed at the four corners of the mobile robot, an 8-channel wireless electrometer for signal acquisition, and an internal data transmission module that transmits the data to a computer via Wi-Fi. Control commands are transmitted via Bluetooth to an Arduino microcontroller, which governs the robot’s motion (Fig. 6a). The reversible deployment of the sensor units enables the rapid reconfiguration of the RO-TWSA, allowing the system to adapt seamlessly to different exploration tasks, as demonstrated in the two representative configurations, Deployment 1 (D1) and Deployment 2 (D2) (Fig. 6b). D1 is designed for obstacle avoidance, providing coverage of both the front and top of the robot to maximize detection of floor-level and overhead obstacles, which is essential for navigation in unknown and confined environments. In contrast, D2 is optimized for map scanning, providing a wider perceptible area that enables the system to detect both forward and backward obstacles simultaneously, making it ideal for exploring the size and shape of unknown environments.

We first demonstrate the system’s autonomous obstacle avoidance capability in the D1 configuration in Fig. 6c and Movie S9, where directional tactile feedback enables real-time response to environmental collisions—an essential capability for autonomous robotic navigation. The output voltage signals from the sensor units during the obstacle avoidance process are presented in Fig. 6d. Based on the relationship between the output voltage and deformation, the computer program interprets the whisker deformation and subsequently controls the robot’s movement, thereby achieving responsive and accurate obstacle avoidance.

By switching the RO-TWSA perception system to the D2 configuration, the robot demonstrates the ability to perform autonomous map scanning. As shown in Fig. 6e and f, the robot is placed at an initial random position within the scene and rotates sequentially to detect surrounding obstacles using its sensor units. Upon contacting the boundary, the sensor units acquire surface information by detecting tactile signals generated through physical interaction. Then, the robot reverses to the initial position and rotates to scan the next direction. By correlating the acquired electrical signals with the robot’s movement distance and simulating the process in MATLAB, the shape and scale of the surrounding environment can be initially estimated. Experimental results in both rectangular and triangular enclosures reveal significant differences in geometric reconstructions, demonstrating the potential of the RO-TWSA-based system for exploring unknown environments—a typical application of robot–environment interactions (Movie S10).

## Conclusion

In this study, we present a reconfigurable omnidirectional triboelectric whisker sensor array comprising multiple sensor units as a novel and effective solution to address the challenges of developing versatile sensing interfaces. The combination of an untethered hydro-sealing vacuum sucker and a triboelectric whisker structure enables reliable and reversible deployment of the sensor units to various surfaces while maintaining high precision in omnidirectional force and motion sensing. Incorporating 4 wt% MXene into silicone enhances the open-circuit voltage by 2.05 times, and its integration into a dual-triangle electrode design enables accurate sensing using only two electrodes, achieving a force threshold of 0.024 N and angular resolution of 5°. The macroporous hydrogel exhibits superior water absorption and retention capabilities, while the incorporation of PBP substantially improves the hydrophilicity of the suction cup base; their synergistic effect enables the UHSVS to maintain reliable multi-surface liquid-sealed adsorption for the sensor units without external water supply. The sensor units exhibit excellent durability, maintaining stable performance after 50,000 sensing cycles and 200 anchoring tests.

Such RO-TWSA has been validated through successful implementation in multiple human–machine–environment interaction scenarios—such as easily deployed teleoperation (human–environment interaction), resolution-adjustable robotic arm palpation (human–robot interaction), and function-switchable robotic autonomous environmental exploration (robot–environment interaction)—demonstrating its high reliability, multi-functionality, and broad applicability. In the future, the RO-TWSA opens promising avenues for its integration into robotics, wearable systems, and complex environments, providing effective solutions for flexible, portable, and reconfigurable interaction interfaces that push the boundaries of adaptive tactile intelligence in complex and dynamic scenarios.

## Experimental Section

### Fabrication of the Sensor Unit

The sensor unit consists of two components: the TWS at the top and the UHSVS at the bottom. These two parts were tightly bonded by a silicone adhesive (Sil-Poxy). Overall weight of the sensor unit is 4.6 g.

The TWS includes two triangular copper electrodes and an MXene/silicone nanocomposite. The two triangular copper electrodes were custom-designed on a polyimide (PI) flexible substrate and fabricated using the PCB process (Fig. S1). A 0.5-mm-thick carbon fiber rod was inserted into the center of the silicone, at the core of the entire sensor unit. The UHSVS consists of a double suction cup, a PBP-silicone ring, and a highly absorbent hydrogel. The PBP was purchased from Polysciences, Inc., and the double suction cups were purchased from Shenzhen VAC Co., Ltd.

### Fabrication Parameters of the 3D-Printed PLA Mold

The PLA mold used for casting the MXene/silicone nanocomposite was fabricated using a Bambu Lab X1 Carbon 3D printer equipped with a 0.4 mm nozzle. The printing material was Bambu PLA Basic, and the build plate was a textured PEI sheet. Slicing was performed in Bambu Studio with the “0.08 mm High Quality @BBL X1C” profile. Key settings included a layer height of 0.08 mm (0.2 mm for the first layer) and 100% infill to ensure mold rigidity. Printing speeds were set to 50 mm s^−1^ for the first layer, 60 mm s^−1^ for outer walls, 120 mm s^−1^ for inner walls, and 150 mm s^−1^ for infill.

### Preparation of the MXene/Silicone Nanocomposite

The MXene (Ti_3_C_2_T_*x*_) nanosheets were purchased from Foshan Xinxi Technology Co., Ltd. Initially, the MXene nanosheets were finely ground using a mortar and pestle. Next, parts A and B of Dragon Skin 30 were mixed in equal weights, and the ground MXene nanosheets were added to the liquid silicone rubber and thoroughly mixed. Subsequently, the liquid mixture was degassed in a vacuum chamber for 5 min to ensure the absence of visible bubbles. The mixture was then poured into a PLA mold fabricated by a 3D printer. Following this, the mixture was baked in an oven at 35 °C for 5 h. Finally, the mold was disassembled to obtain the cured MXene/silicone nanocomposite.

### Preparation of the Highly Absorbent Hydrogel

The crab shell was treated with a saturated hydrochloric acid solution to remove excess calcium carbonate and subsequently treated with a 1.0 mol L^−1^ sodium hydroxide solution to eliminate residual proteins. Following this, deacetylation was performed by heating a 33% sodium hydroxide solution at 90 °C, yielding chitin with a deacetylation degree of 29%. (Detailed explanation is shown in Note S8.) This acid–alkali purification is a standard and widely adopted protocol in chitin/chitosan isolation, ensuring high-purity polysaccharide suitable for nanofiber formation and subsequent hydrogel fabrication [63–65]. Subsequently, the pH of a 0.6 wt% chitin aqueous solution was adjusted to 3 using 99% acetic acid, and the particles were homogenized using a high-speed homogenizer. Finally, the resulting suspension was processed through a high-pressure homogenizer at 600 bars, producing a clear and transparent chitin solution. Preparation of the nanotube solution: Hydroxylated carbon nanotube powder (McLean, China) was dispersed in deionised water and homogenized using a high-pressure homogenizer (600 bar) to obtain a stable aqueous suspension. Chitin nanofibers were prepared based on reported protocols with slight modification [64]. The suspension was first homogenized at 8000 rpm for 3 min using a high-speed homogenizer, followed by high-pressure homogenization at 600 bar for 5 min to obtain nanofibers.

In practical operation, 5 mL of chitin solution was mixed with 50 μL of acetic acid to achieve the desired pH. Then, silk fibroin (SF) was added to the adjusted chitin solution by shaking, resulting in a uniform, cloudy solution without visible precipitate. Following this, the hydroxylated carbon nanotube solution was then added along with the chemical cross-linking agent glutaraldehyde solution, followed by vigorous stirring to ensure thorough and uniform mixing of the solution. The resulting solution was centrifuged at 1500 rpm for 4 min to remove large air bubbles. The homogenized solution was then rapidly transferred into a 3D-printed mold to prevent phase separation due to slow injection speed. The mold was placed in a freezer at − 20 °C for 8 h to allow the hydrogel to fully form. Finally, the frozen hydrogel was removed from the mold, thawed at room temperature in anhydrous ethanol for 3 h to enhance its toughness, and subsequently soaked in deionized water for 1 h to remove residual ethanol (Fig. S12).

### Surface Wettability Characterization of the Hydrogel

The surface wettability of the hydrogel was evaluated by measuring the static water contact angle using a contact angle goniometer (Model: LSA 60). A 5 μL droplet of deionised water was gently deposited onto the hydrogel surface using a microsyringe, and the contact angle was recorded immediately under ambient conditions (~ 25 °C, 50% RH). The measurement was performed within 3 s of droplet placement to minimize evaporation effects. Each sample was tested in triplicate to ensure reproducibility.

### Preparation of the PBP-silicone Ring

First, Parts A and B of the liquid silicone (Ecoflex 00-20) were weighed in a 1:1 mass ratio, with the total mass being *M* g. Then, 6% of *M* g of PBP was weighed and mixed thoroughly with the liquid silicone. Second, the liquid mixture was degassed in a vacuum chamber for 5 min to ensure the absence of visible bubbles. Third, the mixture was poured onto a smooth plastic film, and a 1-mm-thick film was scraped using a film applicator. Fourth, the film was baked in an oven at 35 °C for 5 h. Fifth, after removing the film from the oven, rings with inner and outer diameters of 6 and 15 mm were punched using appropriate punches. Sixth, the rings were carefully detached from the film to obtain the cured PBP-silicone rings. Finally, silicone adhesive (Sil-Poxy) was applied to attach the ring to the upper surface of the underside of the double suction cup.

### Experimental Process and Measuring Equipment

The surface morphologies of the MXene nanosheets and the highly absorbent hydrogel were characterized using a field emission scanning electron microscope (SU-8010, Hitachi). When measuring the electrical output of the TWS, an experimental platform was built using a linear motor (LinMot P01–37 × 120-C/C1100), and the picture of the experimental platform is shown in Fig. S2. The wettability between the water and the PBP-silicone was measured using a contact angle instrument (YUNFAN YF03011100105, China). The solvent used for the PBP-silicone contact angle measurement was deionized water, with a droplet of approximately 10 μL applied for each measurement. To ensure the accuracy and reliability of the data, each reported contact angle value represents the average of three independent measurements. For the data in Figs. 2, 4, and 5, the output signals were measured with a Keithley 6514 electrometer, and a NI-6259 data acquisition card was chosen to collect the data from the sensor unit. The MATLAB interface in LABVIEW was used to process and display the real-time signals measured with the electrometer. For the data of Fig. 6, the output signals were measured with an 8-channel wireless electrometer (internal resistance 1G, sampling capacitance 300 pf), and the signals were sent to a computer in real time (with a sampling frequency of 500 Hz) via Wi-Fi. During demonstration of the potential applications of the RO-TWSA, an Arduino Uno R3 was used to control the LED lights and the movement of the car.

### Texture Recognition Based on the Sensor Unit

The sensor unit was mounted at the end of a robotic arm (C5, JAKA), which slid horizontally at a constant speed of 30 mm/s. These modules (60 × 15 mm^2^) were mounted into a customizable 3D-printed base plate (220 × 220 mm^2^) containing grooves that match the module dimensions, allowing easy interchangeability.

For dataset generation, the robotic arm slid the sensor unit across each texture sample 100 times, capturing a 2-s signal per trial (1 s from each electrode). In total, 4000 s of signal data was collected and divided into a training set (75%) and a test set (25%). The CNN architecture consisted of a Conv1D layer, a MaxPooling layer, a Flatten layer, and a Dense layer. The Conv1D layer employed a convolutional kernel size of 1 × 3, with 8 convolutional kernels and a ReLU activation function. The MaxPooling layer used a pooling window size of 1 × 2. The Flatten layer was applied to flatten the results of the convolution process. The Dense layer consisted of 64 neurons with a ReLU activation function. The final output layer mapped 64 dimensions to 20 dimensions, using the Softmax activation function to perform the multi-classification task (Fig. S24).

The confusion matrix presented in Fig. 5c illustrates the comparison between the model’s predictions and the actual labels, enabling a more intuitive assessment of the model’s classification performance. For example, the first column indicates that, among all samples predicted by the model as class A3, 96% are correctly classified as class A3, whereas 4% are misclassified as class T3. This allows for a direct evaluation of the model’s classification accuracy based on the diagonal elements.

## Supplementary Information

Below is the link to the electronic supplementary material.

Supplementary file1 (MP4 1429 kb)

Supplementary file2 (MP4 2840 kb)

Supplementary file3 (MP4 2757 kb)

Supplementary file4 (MP4 1429 kb)

Supplementary file5 (MP4 2687 kb)

Supplementary file6 (MP4 2093 kb)

Supplementary file7 (MP4 1346 kb)

Supplementary file8 (MP4 1461 kb)

Supplementary file9 (MP4 1522 kb)

Supplementary file10 (MP4 2567 kb)

Supplementary file11 (DOC 19148 kb)
