# Supplementary material for: A Reconfigurable Omnidirectional Triboelectric Whisker Sensor Array for Versatile Human–Machine–Environment Interaction
Source: Nanomicro Lett. 2025 Oct 14;18:76. doi: 10.1007/s40820-025-01930-x (PMC12518198; doi:10.1007/s40820-025-01930-x)
Supplement: Supplementary file 11 — Supplementary file11 (DOC 19148 kb) [file 40820_2025_1930_MOESM11_ESM.doc]

Supporting Information for

**A Reconfigurable Omnidirectional Triboelectric Whisker Sensor Array for Versatile Human-Machine-Environment Interaction**

Weichen Wang^1^, *^#^*, Jiaqi Zhu^1, 2^, *^#^*, Hongfa Zhao^1^, *^#^*, Fei Yao^3^, Yuzhu Zhang^1^, Xiankuan Qian^1^, Mingrui Shu^1^, Zhigang Wu^2^, Minyi Xu^4^, Hongya Geng^3^,*, Wenbo Ding^1^,*, and Juntian Qu^1^,*

^1^ Shenzhen International Graduate School, Tsinghua University, Shenzhen 518055, P. R. China

^2^ State Key Laboratory of Digital Manufacturing Equipment and Technology, Huazhong University of Science and Technology, Wuhan 430074, P. R. China

^3^ Institute of Biomedical and Health Engineering, Shenzhen International Graduate School, Tsinghua University, Shenzhen 518055, P. R. China

^4^ Dalian Key Lab of Marine Micro/Nano Energy and Self-powered System, Dalian Maritime University, Dalian 116026, P. R. China

#Weichen Wang, Jiaqi Zhu, and Hongfa Zhao contributed equally to this work.

*Corresponding authors. E-mail: [geng.hongya@sz.tsinghua.edu.cn](mailto:geng.hongya@sz.tsinghua.edu.cn) (Hongya Geng); [ding.wenbo@sz.tsinghua.edu.cn](mailto:ding.wenbo@sz.tsinghua.edu.cn) (Wenbo Ding); [juntian.qu@sz.tsinghua.edu.cn](mailto:juntian.qu@sz.tsinghua.edu.cn) (Juntian Qu)

**Captions for Videos S1 to S10**

**Video S1** TWS sensitivity testing

**Video S2** The water in the hydrogel is squeezed after 95% of compression

**Video S3** Water-triggered rapid shape recovery of hydrogel

**Video S4** UHSVS anchoring force test and generated force-displacement curve

**Video S5** Application of sensor unit adsorption on different objects

**Video S6** Controlling the on/off state of LEDs

**Video S7** Demonstration of teleoperation enabled by RO-TWSA

**Video S8** Demonstration of resolution-adjustable robotic arm palpation enabled by RO-TWSA

**Video S9** Demonstration of obstacle avoidance enabled by RO-TWSA

**Video S10** Demonstration of map construction enabled by RO-TWSA

**Table S1** Comparative evaluation of performance metrics for the UHSVS and other representative anchoring methods

| Anchoring Method | Actuating Source Independence | Manufacturing Simplicity | Surface Adaptability | Anchor Strength | Structural Simplicity | Portability |
| --- | --- | --- | --- | --- | --- | --- |
| **UHSVS**  ***(This work)*** | **High** | **High** | **Medium** | **High** | **High** | **High** |
| Microspine [S1] | High | Low | Medium | Medium | Medium | High |
| Dry adhesive [S2] | High | Low | Medium | High | Medium | High |
| Electrostatic pad [S3] | Low | Medium | Low | Low | High | Medium |
| Conformal grip actuator [S4] | Low | Medium | Medium | Low | Medium | Medium |
| Vacuum adhesion device [S5] | Low | High | Medium | High | High | Low |

**Table S2** Comparison of features between the sensor unit of RO-TWSA and previously reported whisker sensors

| No. | Year | Sensor Type | Minimum Angular Resolution (°) | Number of Electrodes | Angular Electrodensity Resolution (°) | Force Resolution (N) | Refs. |
| --- | --- | --- | --- | --- | --- | --- | --- |
| **1** | **2025** | **Triboelectric** | **5** | **2** | **10** | **0.024** | ***This work*** |
| 2 | 2024 | Resistive | 45 | 4 | 180 | 0.0024 | [S6] |
| 3 | 2019 | Resistive | 15 | 4 | 60 | 0.002 | [S7] |
| 4 | 2018 | Resistive | 180 | 1 | 180 | 0.012 | [S8] |
| 5 | 2019 | Magnetic | 0.7 | 4 | 2.8 | 0.2 | [S9] |
| 6 | 2024 | Magnetic | 180 | 1 | 180 | 0.005 | [S10] |
| 7 | 2023 | Piezoelectric | 180 | 1 | 180 | 0.00007 | [S11] |
| 8 | 2024 | Piezoelectric | 180 | 1 | 180 | 0.0013 | [S12] |
| 9 | 2023 | Triboelectric | 360 | 1 | 360 | 0.01 | [S13] |
| 10 | 2024 | Triboelectric | 180 | 2 | 360 | 0.05 | [S14] |
| 11 | 2021 | Triboelectric | 180 | 2 | 360 | 0.0002 | [S15] |
| 12 | 2021 | Triboelectric | 30 | 4 | 120 | 0.28 | [S16] |
| 13 | 2022 | Triboelectric | 30 | 4 | 120 | 0.16 | [S17] |
| 14 | 2022 | Triboelectric | 18 | 2 | 36 | 0.1 | [S18] |

Angular electrodensity resolution= Minimum angular resolution*Number of electrodes

**Supporting Notes**

**Note S1** **Functional role of the carbon fiber rod**

The carbon fiber rod serves as an artificial vibrissa (whisker), playing a central role in mechanical sensing. Its primary function is to transmit mechanical deformation resulting from contact or collision with external objects to the MXene/silicone nanocomposite, thereby generating electrical signals for omnidirectional tactile perception.

The carbon fiber material was chosen after comparative analysis with other alternatives reported in literature. For example:

Xu et al. [S16] developed whisker sensors using Ti-Ni shape memory alloy wires, but still differ significantly in flexibility and compliance from biological whiskers.

Hou et al. [S19] utilized plastic optical fibers, which suffer from mechanical fatigue and poor resistance to temperature and UV exposure.

By contrast, carbon fiber rods combine high flexibility, light weight, and excellent mechanical stability, making them ideal for fabricating artificial whiskers that need to endure high-frequency contact and large deformation. Their stiffness and resilience allow the TWS to respond sensitively to directional forces while maintaining structural durability across repetitive cycles.

In summary, the carbon fiber rod offers an optimal balance between biomimetic functionality, mechanical performance, and fabrication feasibility, which makes it well-suited for the whisker element of the TWS.

**Note S2 Dynamic model of the TWS**

The triboelectric whisker structure is composed of a carbon fiber rod and a silicone rod, and the whole presents an elongated morphology, which is in line with the basic assumption of the Euler-Bernoulli beam theory, i.e., the length of the beam is much larger than its cross-sectional dimensions, and the effect of shear deformation can be neglected[S6] In the practical application of the TWS, the deformation is usually small and complies with the assumption of geometric linearity, so it is reasonable to adopt the linear elasticity principle relationship to describe its mechanical behaviour. When the whisker is subjected to an external force, its bending response can be described by Eq. S1.

| 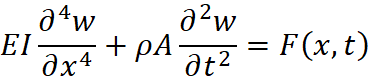 | (S1) |
| --- | --- |

where
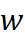
 denotes the transverse displacement of the beam,
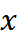
 is the axial position of the beam, and
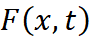
 is the external load acting on the beam at time
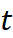
.
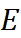
 is the modulus of elasticity,
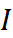
 is the moment of inertia of the cross-section,
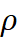
 is the density of the carbon fibre rod and
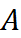
 is the cross-sectional area of the beam. By equating the silicone flexible rod to a linear spring and setting the position of the linear spring at the end of the silicone rod,
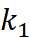
 and
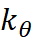
 can be analytically derived to characterize the linear and torsional stiffness of the silicone rod.

Since the force on the part of the elastic rod embedded in the silicone rod can be regarded as a distributed load
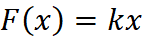
, where
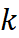
 is the slope, the total force on the silicone rod
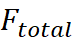
 can be calculated by integration:

| 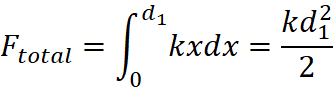 | (S2) |
| --- | --- |

where
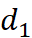
 is the length of the silicone rod. Under the distributed load, the bending differential equation of this elastic rod can be obtained:

| 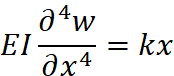 | (S3) |
| --- | --- |

Based on the boundary condition, at
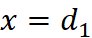
, the elastic rod is subjected to both shear force and bending moment of 0. At
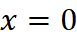
, the base slope and displacement of the elastic rod are 0, and the expression for displacement
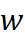
 is obtained:

| 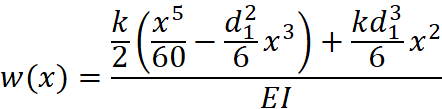 | (S4) |
| --- | --- |

*Based on the equivalent spring at the force generated should be equal to the actual distributed load
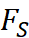
, and
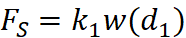
*

| 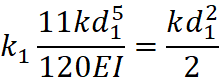 | (S5) |
| --- | --- |

*Solution:*

| 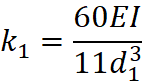 | (S6) |
| --- | --- |

The mechanical behaviour of the system can be described by the load balance equation with respect to and expressions as follows:

| 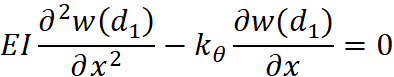 | (S7) |
| --- | --- |
| 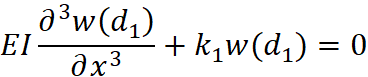 | (S8) |
| 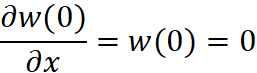 | (S9) |
| 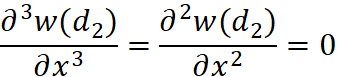 | (S10) |

where
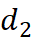
 is the length of the carbon fibre rod.


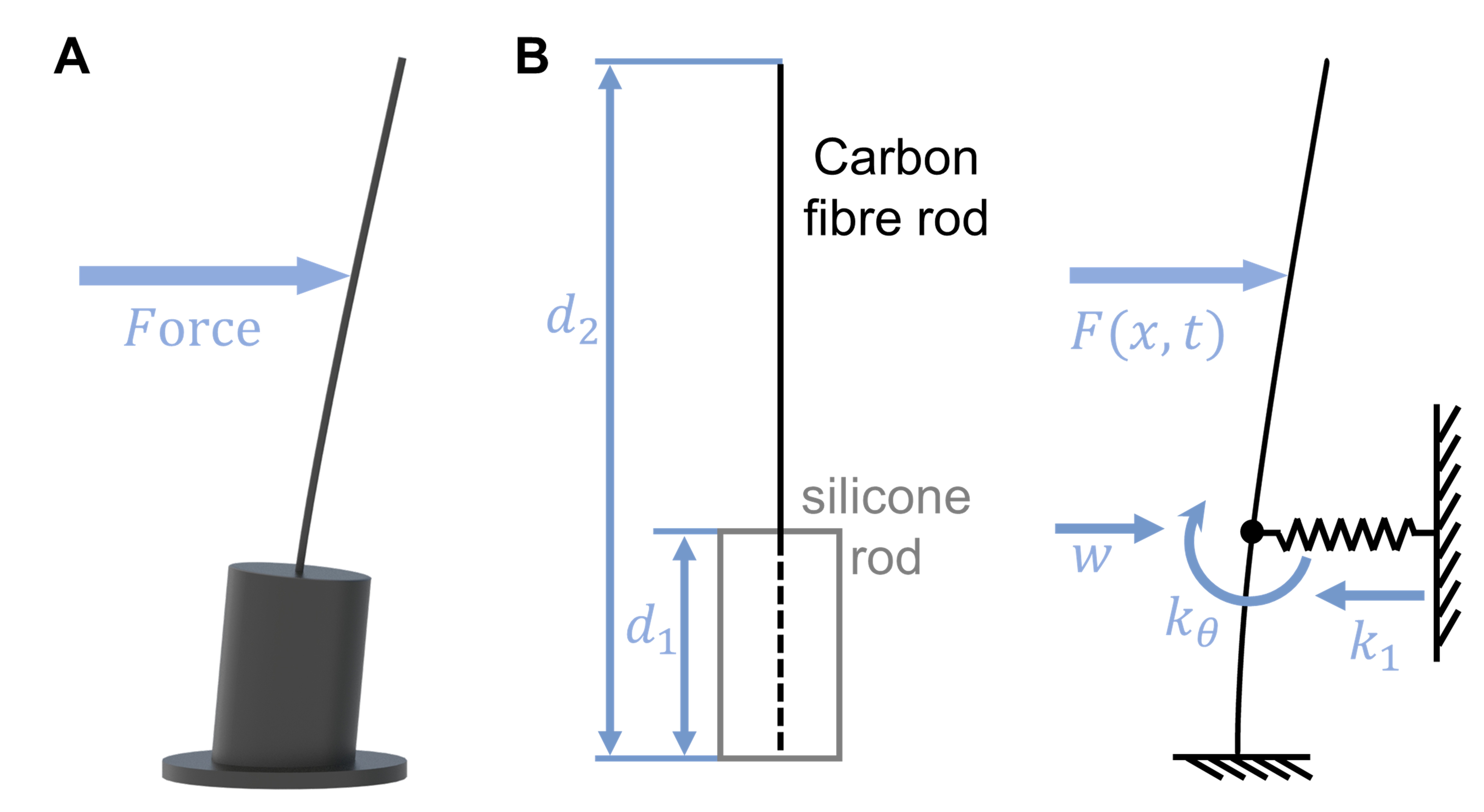


**Fig. N1** **A** Schematic diagram of the external force applied to the TWS. **B** A simple ensemble parametric model to describe the dynamics of the TWS. The silicone rod is described by a torsion spring (
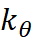
) and a linear spring
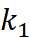
 at
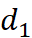
.
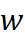
 is the beam lateral displacement at the silicone rod.

**Note S3 COMSOL simulation for the potential distribution**

A 3D multiphysics model was constructed based on the sensing mechanism of TWS. The simulation involved the following modules and steps:

1. Modules used:

The simulation employed the AC/DC Module, Solid Mechanics Module, and the CAD Import Module, under the “General Study > Stationary” setting.

2. Model construction:

The geometry of the TWS—including the MXene/silicone nanocomposite, copper electrodes, a carbon fiber rod, and PI substrate—was designed in SolidWorks and imported into COMSOL. A large cubic domain was added around the sensor to simulate an infinite air region.

3. Material settings:

Five materials were assigned: MXene/silicone nanocomposite, carbon fiber rod, copper electrodes, PI substrate, and surrounding air. For each material, Young’s modulus, Poisson’s ratio, and relative permittivity (dielectric constant) were manually defined in the "User-Defined Materials" section.

4. Boundary and electrical conditions:

1) The large air domain was grounded and set to zero net charge.

2) Both upper and lower triangular copper electrodes were assigned surface charges of +Qc and the silicone layer was assigned a net charge of −2Qc.

3) In the solid mechanics module, the bottom surfaces of the PI substrate, the bottom surfaces of the silicone rod, and the carbon fiber rod were all set as fixed constraints to ensure mechanical stability. A boundary load was applied to the carbon fiber rod to simulate external forces.

5. Mesh and physics:

1) The mesh size was set to “Normal”.

2) The sidewall of the MXene/silicone nanocomposite was meshed using mapped mesh, while the rest used free tetrahedral mesh.

3) Three physical interfaces were selected: Electrostatics, Solid Mechanics, and Moving Mesh.

6. Simulation goal:

The model solved for the potential difference between the two electrodes and the MXene/silicone nanocomposite under varying external forces and directional inputs. This enabled visualization of electric potential gradients and the piezoelectric-electric coupling behavior in the TWS.

**Note S4 Explanation of TWS high sensitivity**

The characterization of “high sensitivity” in our work is based on both force magnitude resolution (0.024 N) and angular resolution (5° with 2 electrodes, i.e., 10° angular electrodensity), as outlined in Supplementary Table S2. This dual-axis sensitivity—force magnitude and direction—is critical for whisker-based tactile sensing in unstructured environments.

While some reported sensors have slightly lower force resolution (e.g., 0.002–0.01 N), they often suffer from poor angular resolution (typically 180° with one electrode, angular electrodensity = 180°) [S8, S12, S14]. In contrast, our sensor combines millinewton-level force detection with high angular electrodensity resolution, enabling more precise mechanical feedback for robotic perception tasks.

Therefore, the term “high sensitivity” in our manuscript is contextually justified, reflecting the combined performance in both directional and magnitude sensing.

**Note S5 Relatively large pore size of hydrogel**

To characterize the porous structure of the hydrogel, we performed scanning electron microscopy (SEM) on freeze-dried hydrogels. The freeze-casting process produces elongated, aligned channel-like pores rather than isolated spherical cavities according to our previous report [S20, S21]. Therefore, we estimated porosity using the area-fraction method on cross-sectional SEM images (ImageJ segmentation), which is an accepted and widely used proxy for volumetric porosity when pores are predominantly through-pores aligned with the freezing direction (in that case 2D area fraction ≈ 3D volume fraction). Using this approach with the measured mean pore diameter (D) and wall thickness (w), and assuming close hexagonal packing of cylindrical/columnar pores in cross section, the porosity is given by:


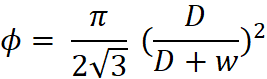


which yields ϕ = 76.2%, substituting D = 165 μm and w = 15 μm. This value is consistent with the strongly open, channel-like architecture visible in the SEM images and is comparable with previous reports, such as a super microporous hydrogels consisting of polyvinyl alcohol (PVA) and sodium alginate [S22].

Additionally, the relatively large pore size (~165 μm) observed here is consistent with our previous reports of chitosan-based gels [S23–S25]. This is primarily attributed to the semi-rigid nature of the chitosan polysaccharide chains and their extensive hydrogen bonding, which together exert only a limited inhibitory effect on ice crystal growth, thereby allowing the formation of comparatively large ice crystals. Upon freeze-drying, these crystals are removed, leaving behind elongated, channel-like pores whose diameter closely reflects the original ice crystal dimensions. Additionally, the low polymer solid content and the freezing rate used in our fabrication promote the growth of larger ice crystals, further contributing to the observed pore size.

**Note S6 Reasons for choosing PBP**

While hydrophilicity is indeed a fundamental driver behind the use of PBP in UHSVS (Fig. 3g), it is not the sole reason for its selection. Below, we elaborate on additional critical considerations that guided this material choice:

1. **Compatibility with Silicone:**

One of the essential requirements for the UHSVS design is maintaining structural and interfacial uniformity between the hydrophilic component and the silicone substrate. PBP, as a block copolymer with a siloxane segment, exhibits excellent miscibility and chemical compatibility with standard silicone. This compatibility allows PBP to be uniformly incorporated into the silicone network, avoiding undesirable phase separation and ensuring consistent material integrity throughout the PBP-silicone ring [S26].

2. **Minimal Impact on Mechanical Properties:**

The suction performance depends not only on hydrophilicity but also on the mechanical properties of the material. Our experiments showed that incorporating PBP does not significantly compromise the elasticity, softness, or rebound capability of the base silicone [S27]. These properties are critical for enabling the reversible deformation and recovery of the UHSVS under cyclic loading. In contrast, other hydrophilic additives often increase stiffness or brittleness, which undermines the sealing and reusability of the structure.

3. **Cost and Manufacturing Scalability:**

PBP has a relatively established supply chain and can be synthesized or acquired at scale with controllable quality and cost. The process of integrating PBP into silicone using standard casting and curing protocols remains straightforward, thereby supporting repeatable and economical fabrication. These features are advantageous over more exotic or expensive hydrophilic polymers with similar water-affinity profiles.

4. **Stability and Durability:**

PBP-modified silicone demonstrates long-term stability in maintaining water retention and anchoring strength, even after repeated use. Our 200-cycle durability test (Fig. S17) showed minimal degradation in suction force, which we attribute to the stable water-holding and diffusion barrier properties of the PBP-silicone ring. This durability underlines the importance of choosing a copolymer that not only imparts hydrophilicity but also retains function in cyclic mechanical and environmental stresses.

In summary, the selection of PBP was a holistic decision informed by its (i) chemical compatibility with silicone, (ii) negligible effect on essential mechanical characteristics, (iii) cost-effective processing advantages, and (iv) stable performance under repeated actuation—all of which are vital for the operational requirements of RO-TWSA in dynamic, real-world applications.

**Note S7 Confusion matrix of texture classification**

The convolutional neural network (CNN) used in this work was implemented using the PyTorch framework in Python, and both its training and deployment were conducted on a hardware platform equipped with NVIDIA GeForce RTX 4090 ×3 hardware. The structure of the CNN model is detailed as follows:

| class CNN(nn.Module):  def __init__(self, input_channels, input_length):  super(SimpleCNN, self).__init__()  #input_channels：the number of input modalities  #input_length：the data length of a single modality  # Convolutional layer: input_channels -> 8 output channels, kernel size = 3  self.conv1 = nn.Conv1d(in_channels=input_channels, out_channels=8, kernel_size=3)  # Max pooling layer: window size = 2  self.pool = nn.MaxPool1d(kernel_size=2)  # Automatically compute the flattened feature dimension after convolution and pooling  conv_output_length = (input_length - 3 + 1) // 2 # After conv, then pooling  self.flattened_size = conv_output_length * 8  # Fully connected layer with 64 neurons  self.fc1 = nn.Linear(self.flattened_size, 64)  # Output layer: 20 classes  self.fc2 = nn.Linear(64, 20)    def forward(self, x):  # x shape: (batch_size, input_channels, input_length)  x = F.relu(self.conv1(x))  x = self.pool(x)  x = x.view(x.size(0), -1) # Flatten  x = F.relu(self.fc1(x))  x = F.softmax(self.fc2(x), dim=1) # Multi-class classification  return x |
| --- |

**Note S8 Degree of deacetylation (DDA) of chitosan**

The degree of deacetylation (DDA) of chitosan was determined using **^1^H NMR spectroscopy** in **deuterated trifluoroacetic acid (CF_3_CO_2_D, TFA-d)**. The DDA was calculated based on the **integral ratio method**, where **H1D** represents the integral area of the anomeric proton of the deacetylated unit (D-glucosamine) appearing at **δ ≈ 6.0–6.3 ppm**, and **HAc** corresponds to the methyl protons of the acetyl group (–COCH_3_) in the acetylated unit (N-acetyl-D-glucosamine), observed at **δ ≈ 3.0–3.2 ppm**. This calculation method is widely accepted for quantifying DDA in partially deacetylated chitin.

For convenience, we have displayed the additive calculation details below:

The degree of deacetylation (DDA) was calculated from the ¹H NMR spectrum in deuterated trifluoroacetic acid (CF_3_CO_2_D) using the integral ratio method:

| 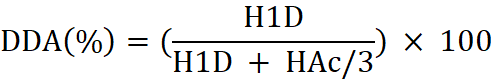 | (S11) |
| --- | --- |

where **H1D** is the integral of the anomeric proton of the deacetylated unit (δ ≈ 6.0–6.3 ppm) and **HAc** is the integral of the methyl protons of the acetyl group (δ ≈ 3.0–3.2 ppm).


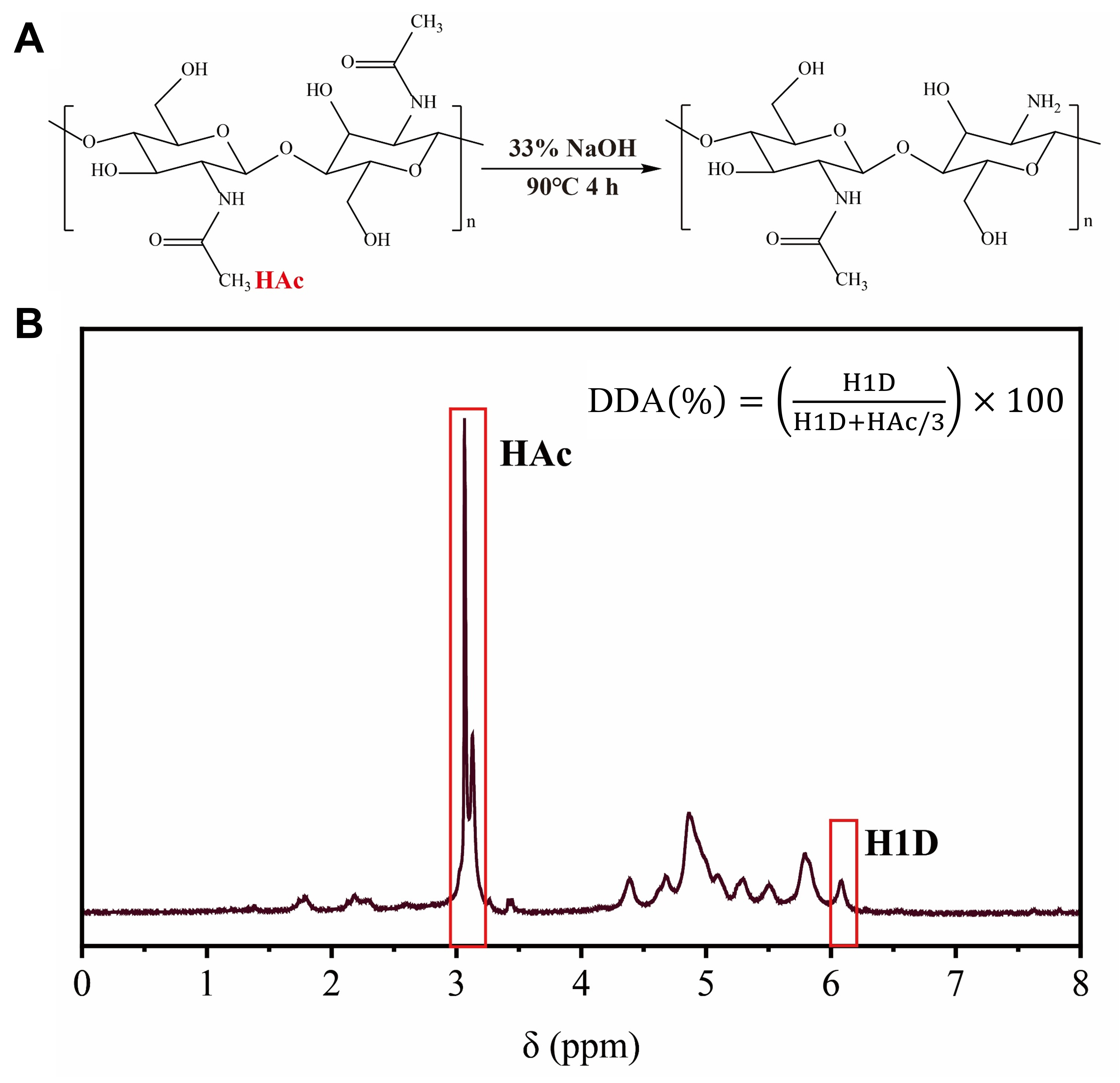


**Fig. N2 A** The chemical reaction of deacetylation and its specific conditions and **B** ^1^H NMR of deacetylated chitin


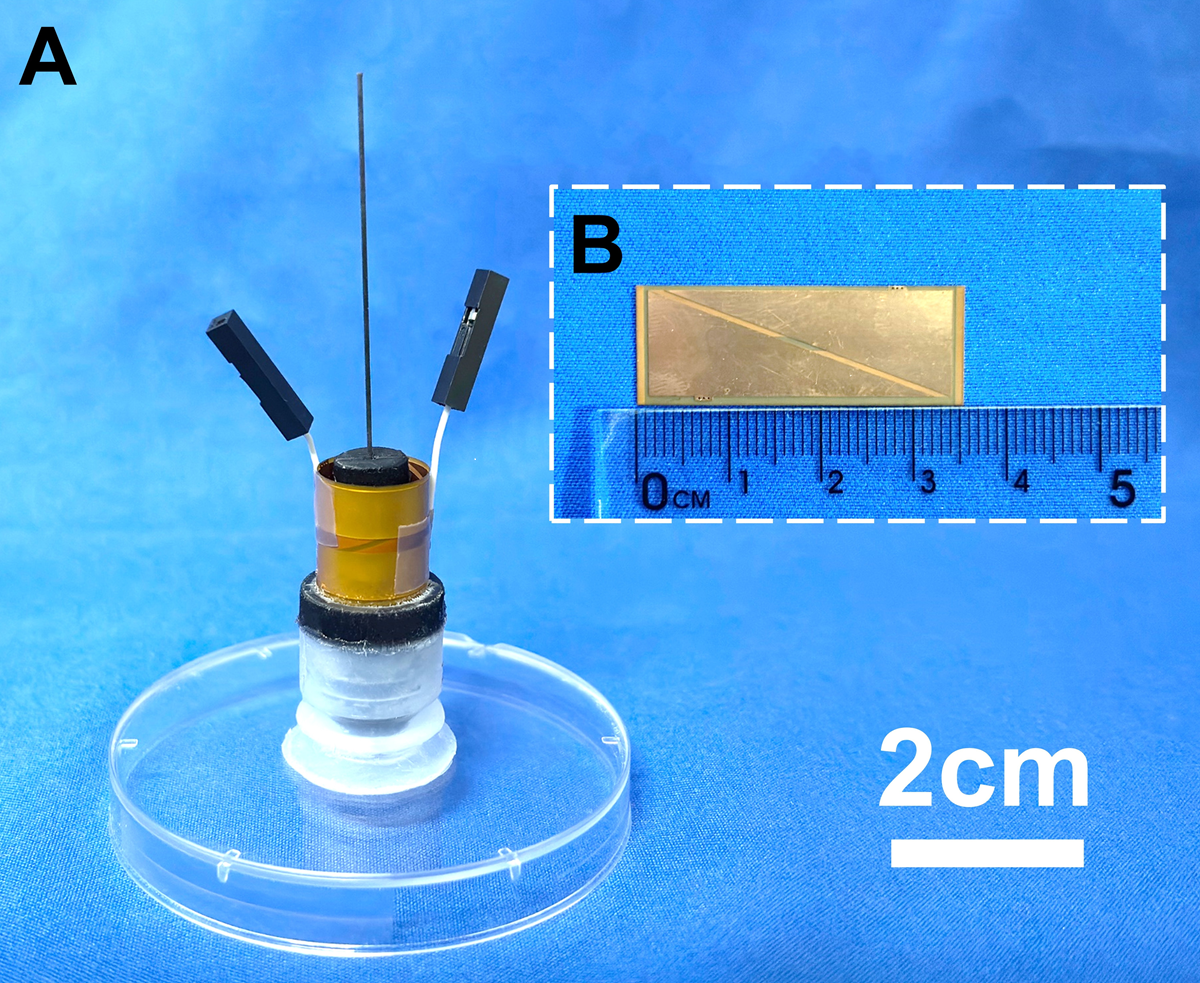


**Fig. S1** Optical image of the sensor unit. (**A**) Overall view of sensor unit. (**B**) Unfolded view of the electrode


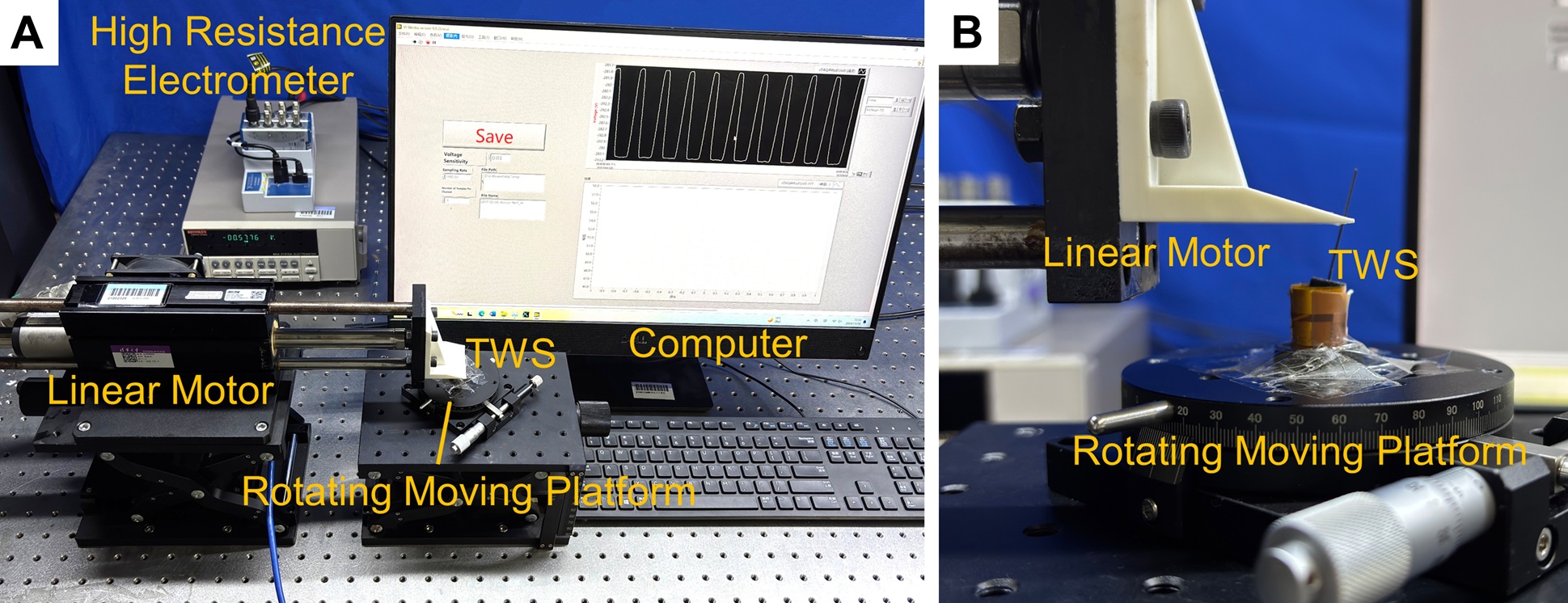


**Fig. S2** Experimental setup for TWS testing. **A** Overview of the experimental setup. **B** Close-up of the TWS positioned on the rotating moving platform


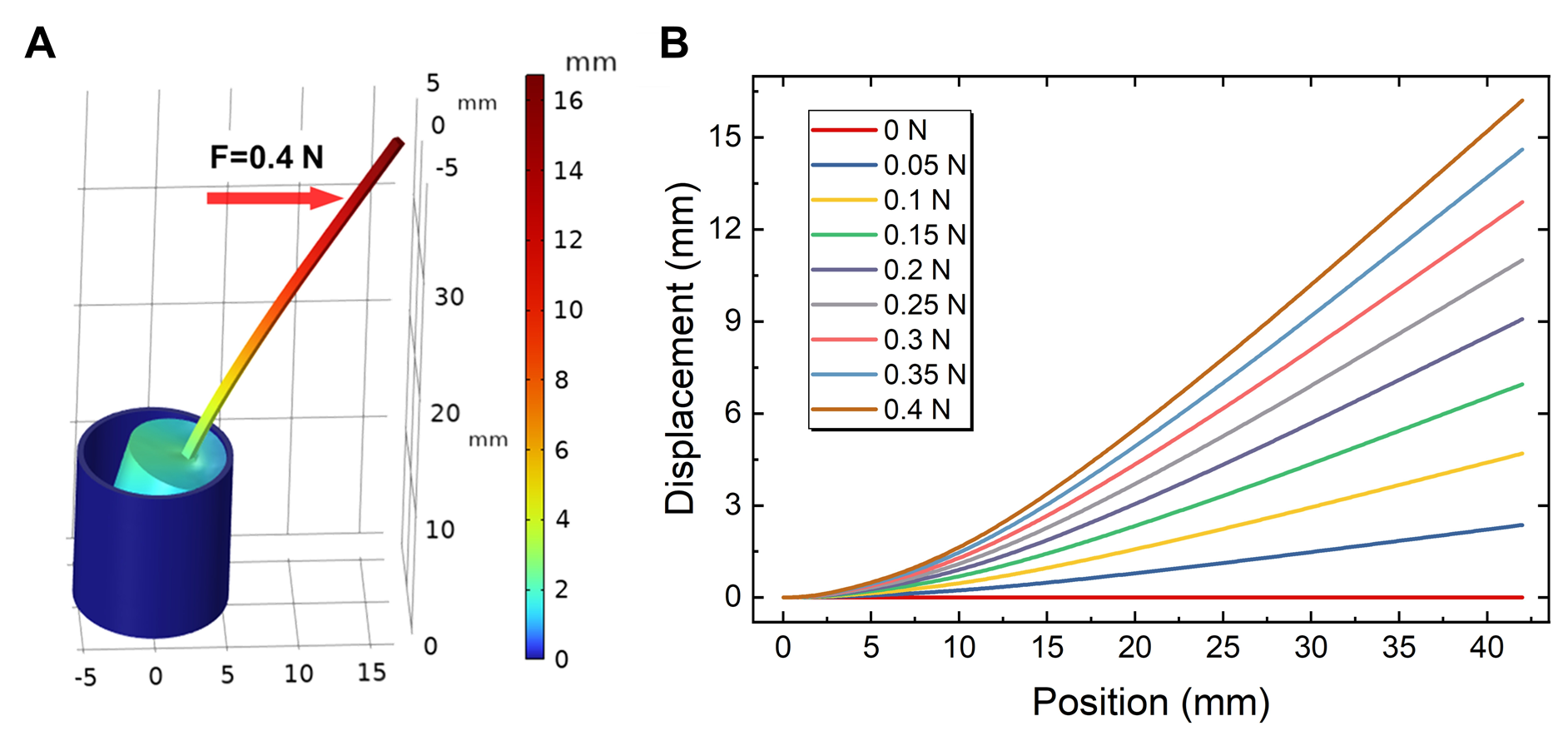


**Fig. S3** Simulated deformation results of the MXene/silicone nanocomposite and carbon fiber rod under applied force. **A** Displacement distributed contour of the MXene/silicone nanocomposite and carbon fiber rod when a force is applied on the carbon fibre rod. **B** Displacements generated at different positions of the MXene/silicone nanocomposite and carbon fibre rod under different applied forces


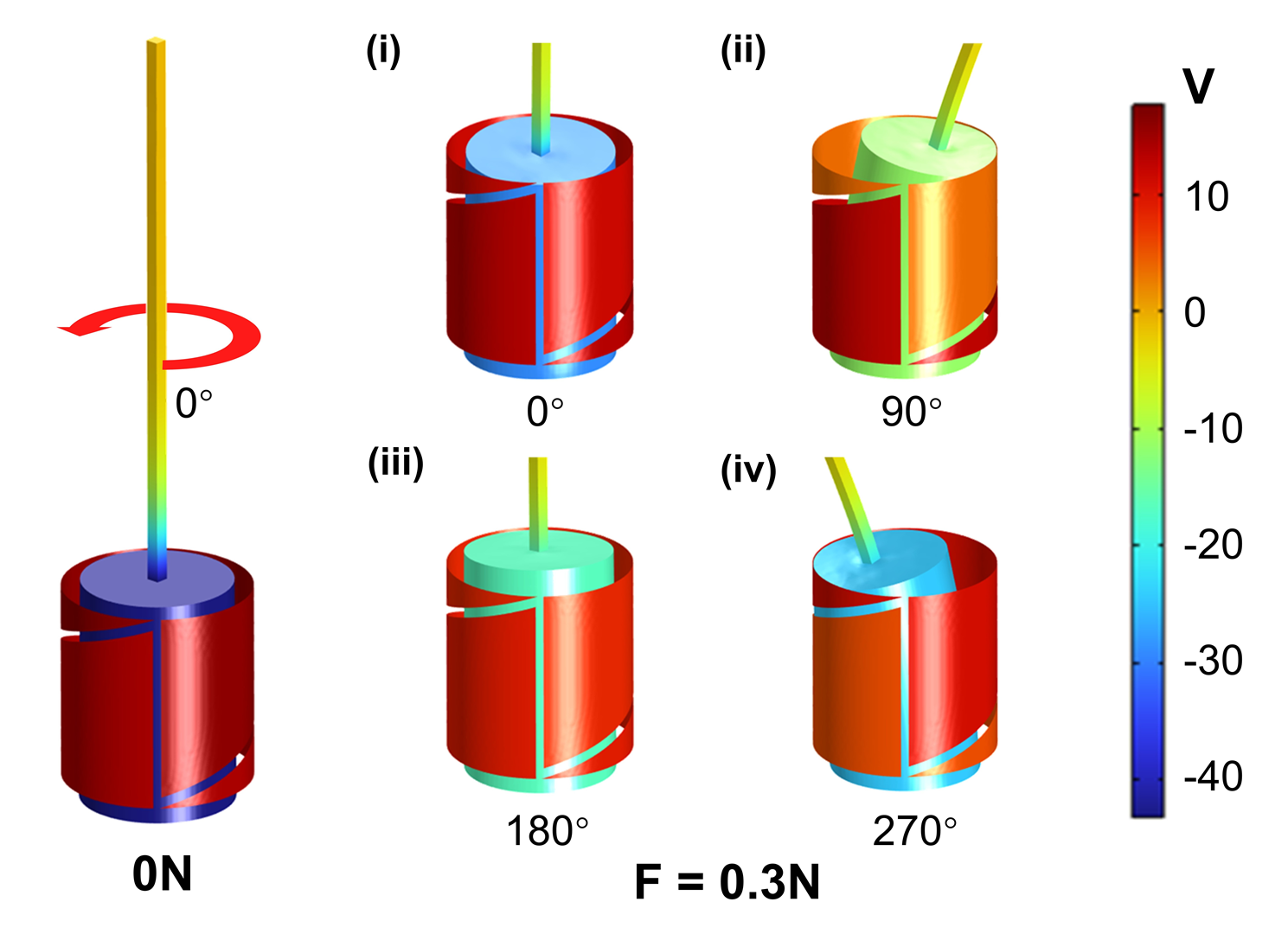


**Fig. S4** COMSOL simulation of potential changes between the two electrodes and the MXene/silicone nanocomposite of the TWS at different compression angles: (i) 0°, (ii) 90°, (iii) 180°, (iv) 270°

**
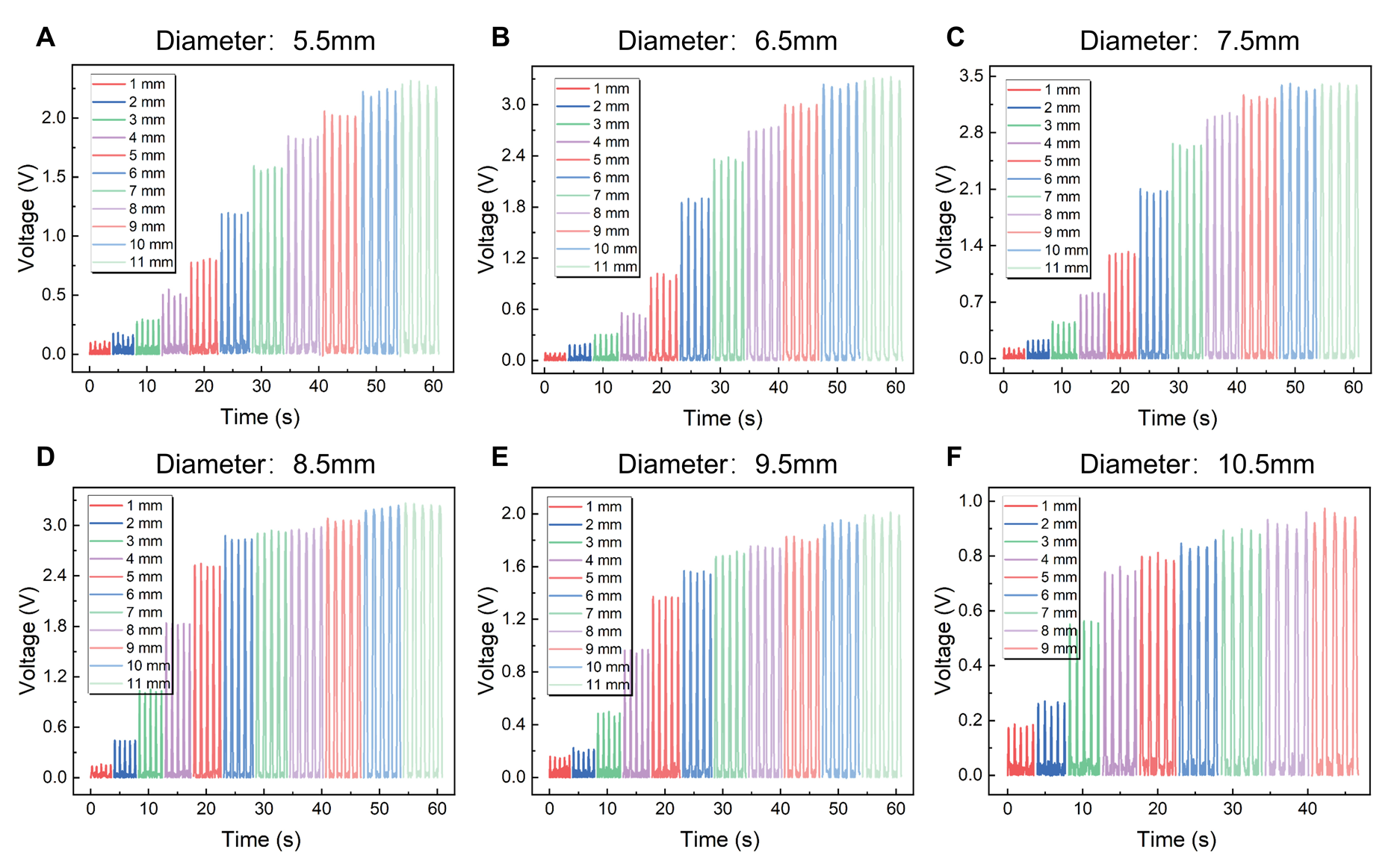
**

**Fig. S5** Voltage response of the TWS with varying MXene/silicone nanocomposite diameters. **A** 5.5mm. **B** 6.5mm. **C** 7.5mm. **D** 8.5mm. **E** 9.5mm. **F** 10.5mm

**
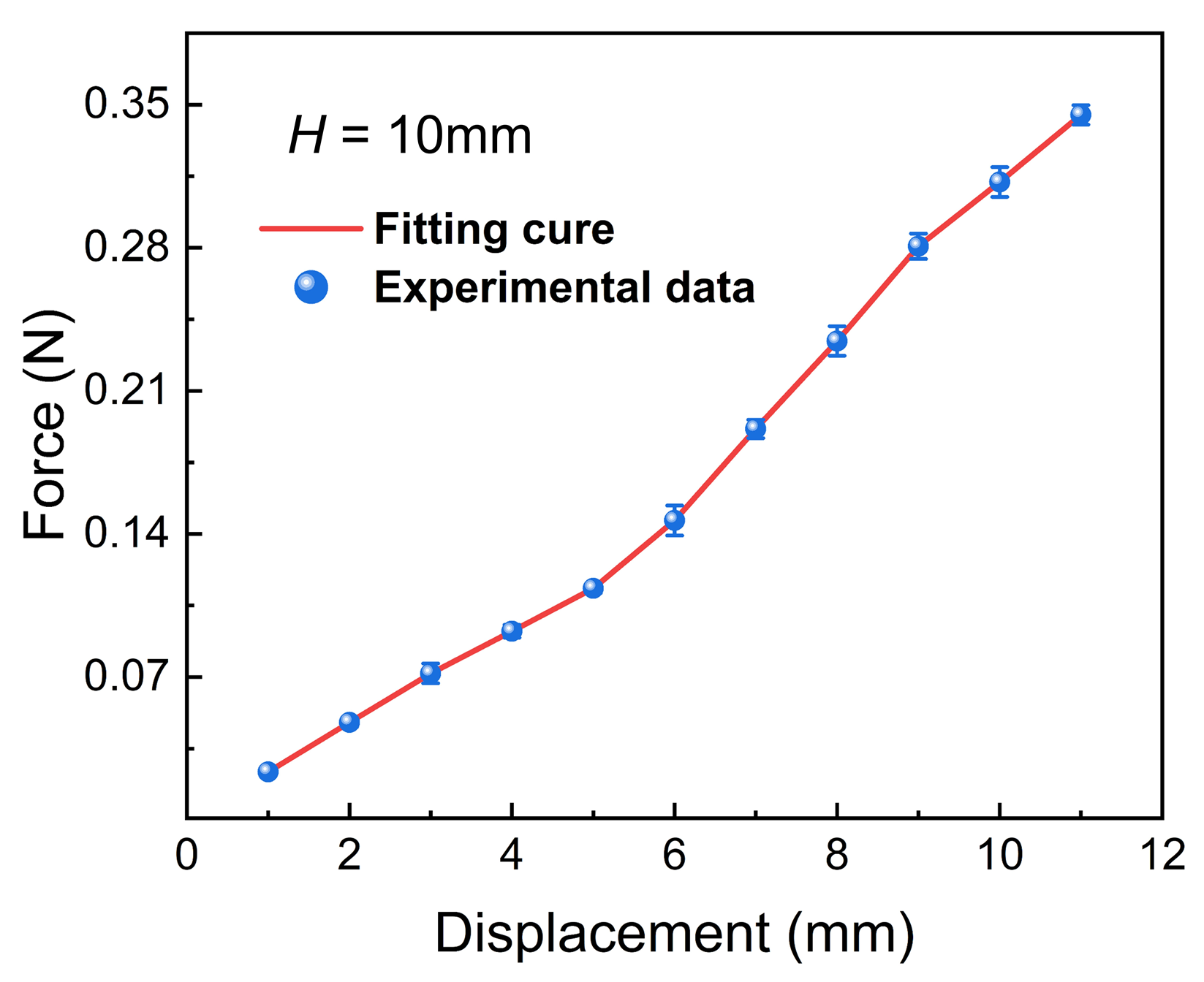
**

**Fig. S6** Force response of the TWS with varying displacements (from 1 to 11 mm)

**
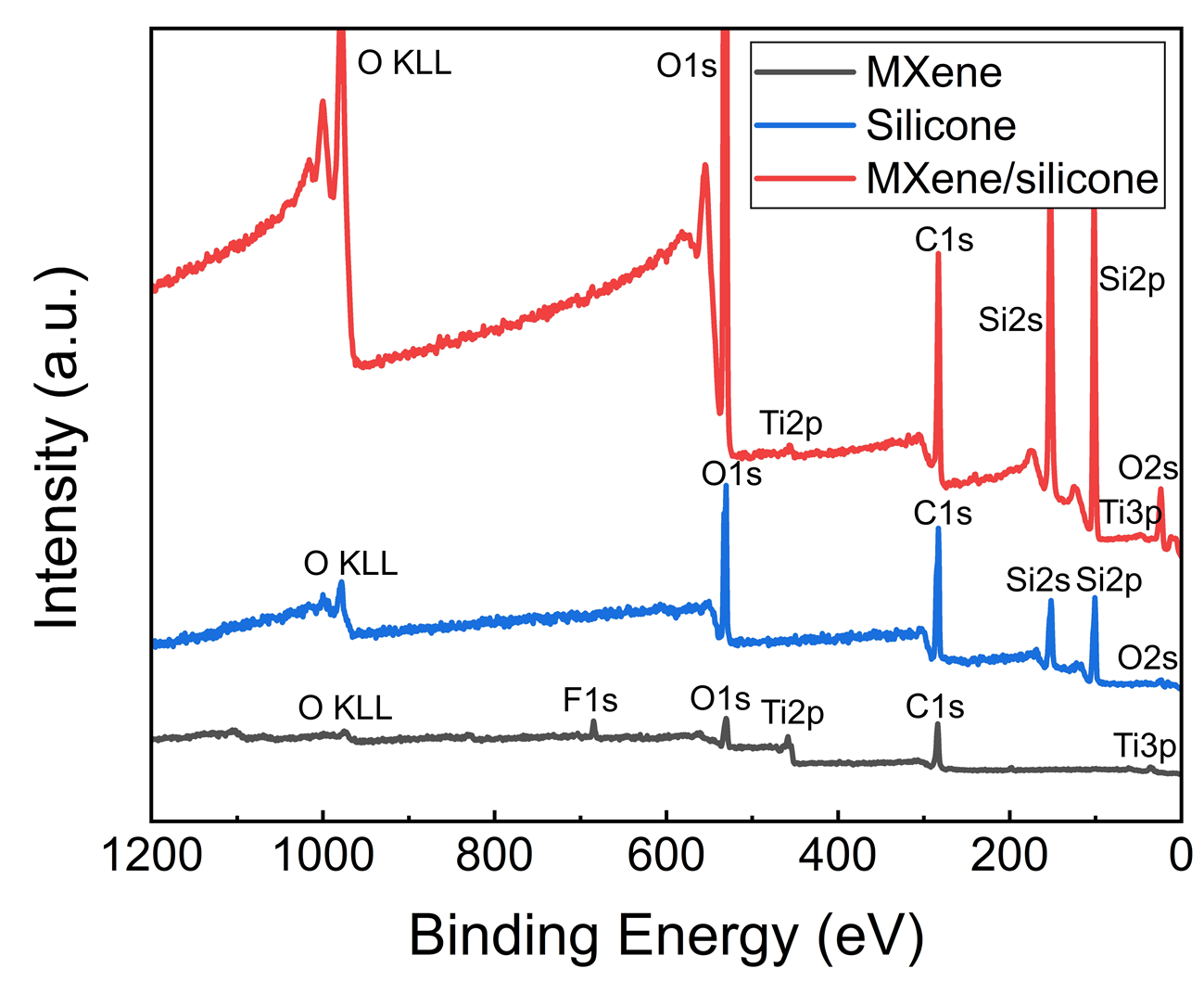
**

**Fig. S7** Survey X-ray photoelectron spectroscopy (XPS) spectra of the MXene, Silicone, and MXene/silicone nanocomposite

**
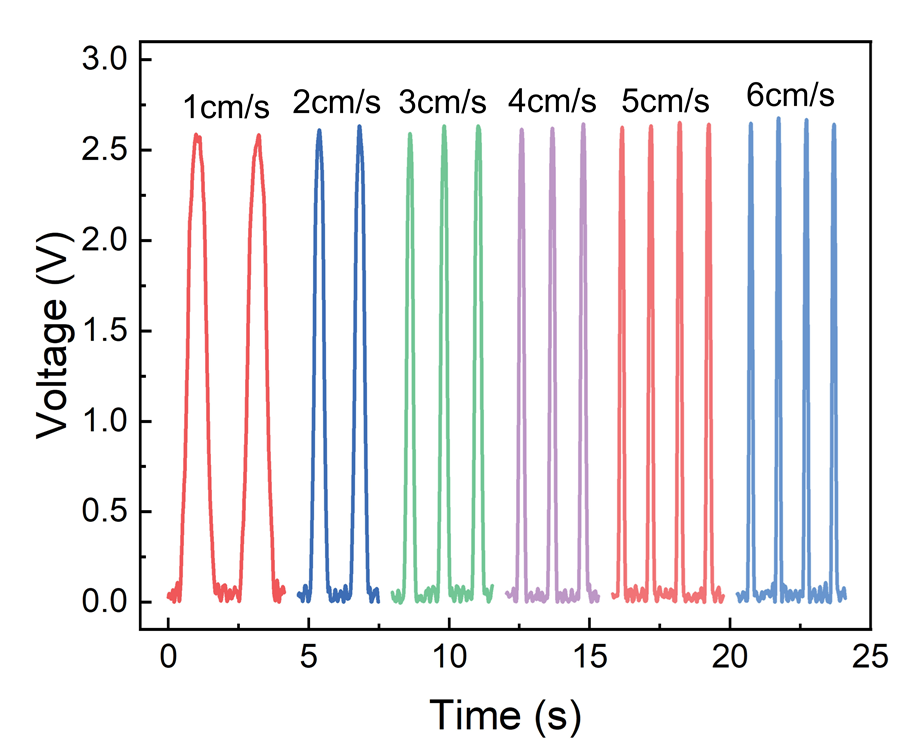
**

**Fig. S8** Response range of voltage to external load velocity from 1 to 6cm s^−1^


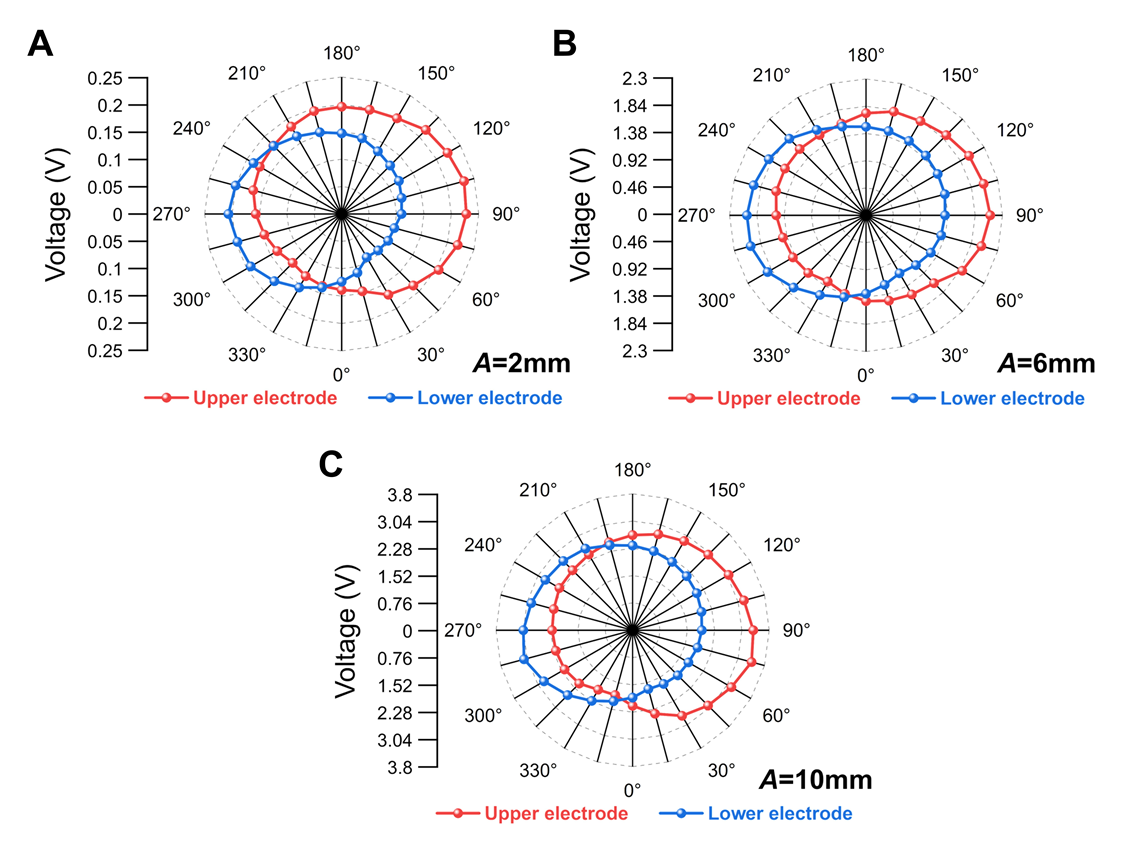


**Fig. S9** The open circuit voltage response to stimuli at varying angles under the conditions of: **A** *A*= 2mm, **B** *A* = 6mm, **C** *A* = 10m

**
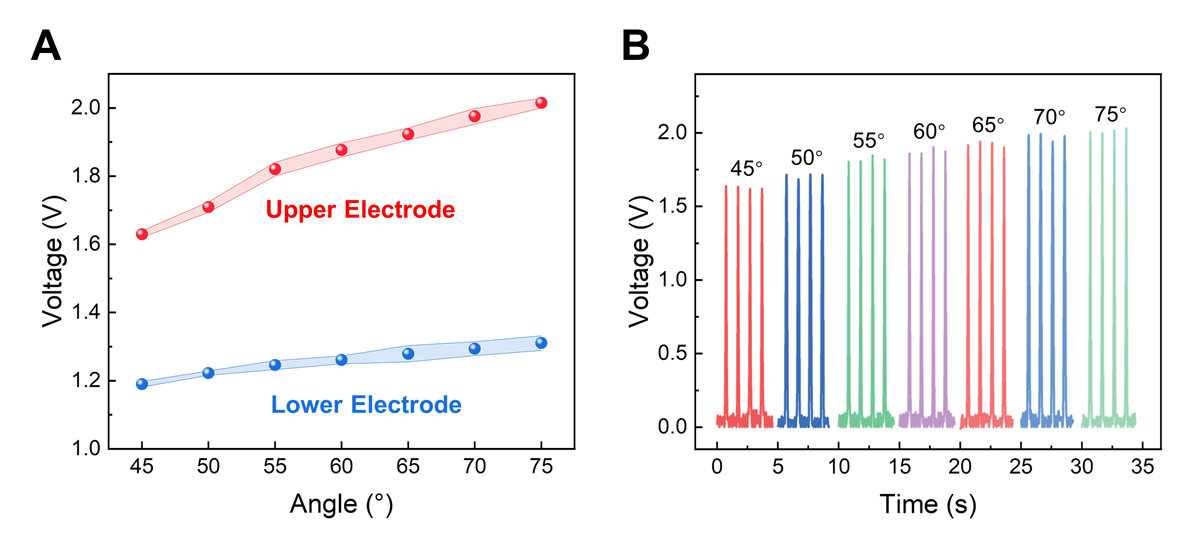
**

**Fig. S10** Resolution of TWS force direction **A** Voltage response of upper and lower electrodes at intervals of 5°. **B** Detailed output of the upper electrode

**
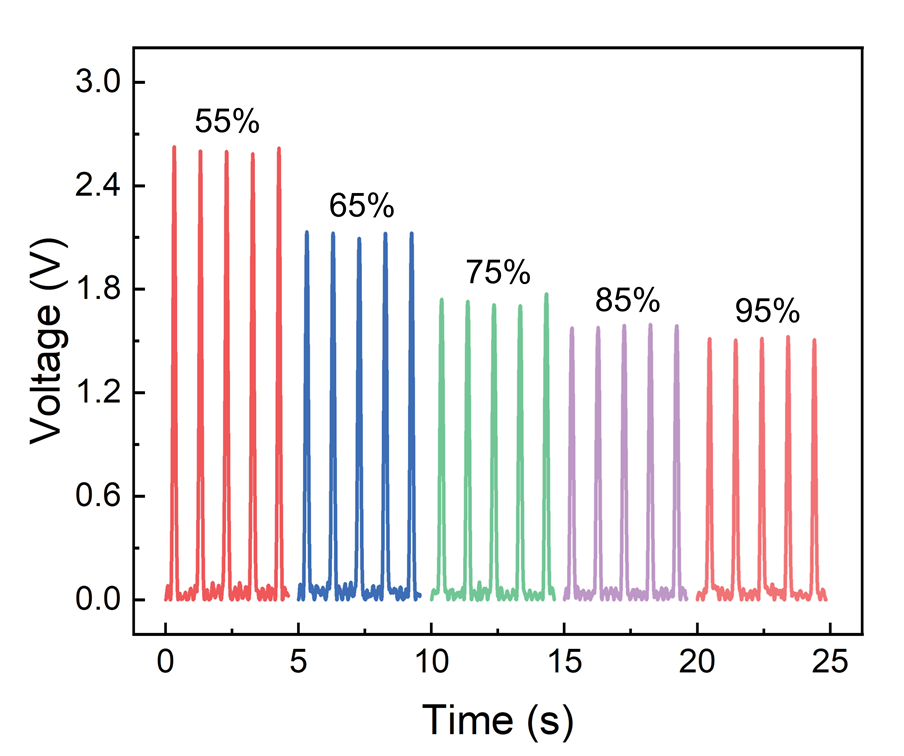
**

**Fig. S11** Variation of open-circuit voltage with humidity

**
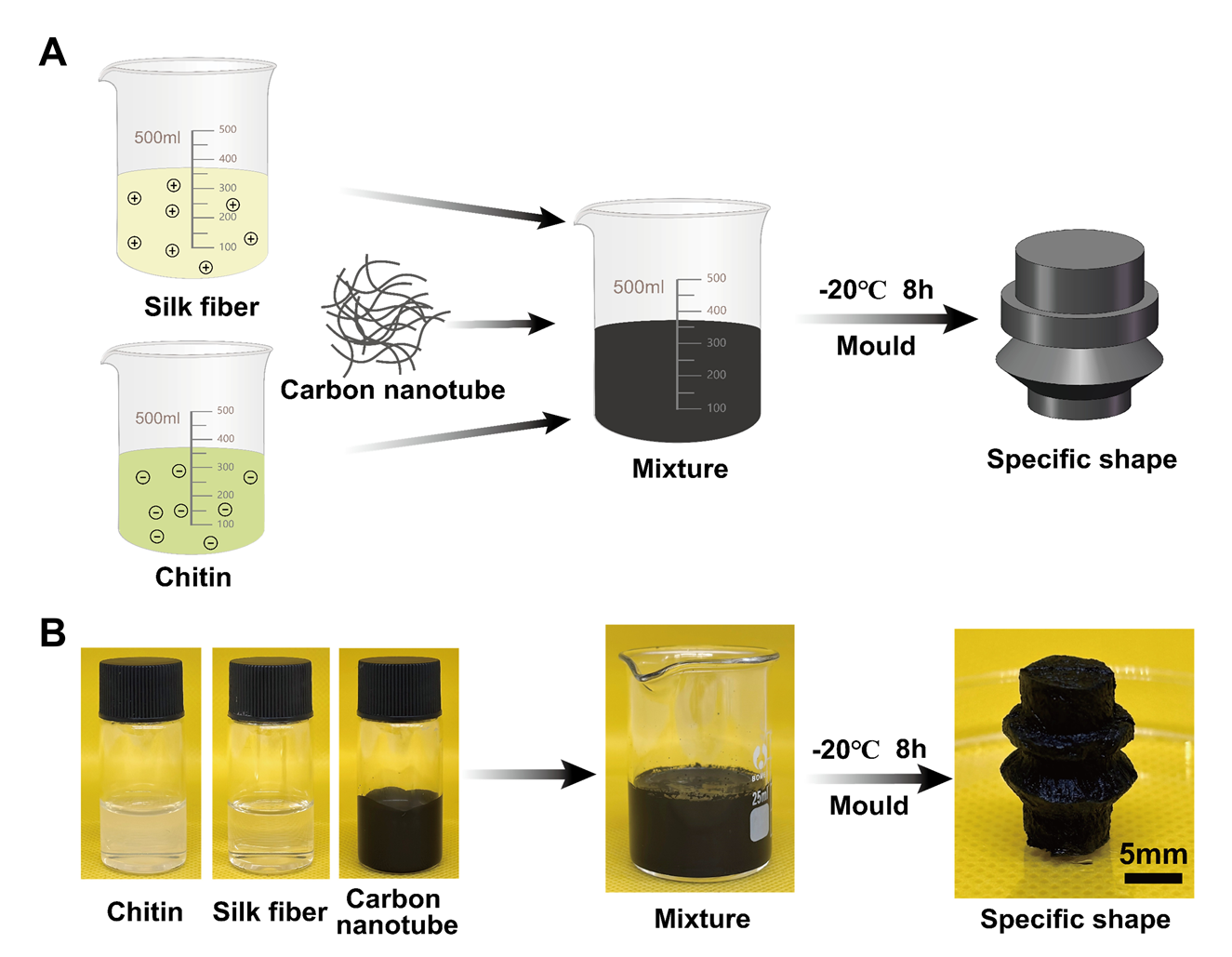
**

**Fig. S12** Fabrication process of the highly absorbent hydrogel. **A** Schematic diagram of the fabrication process. **B** Corresponding physical image of the process

**
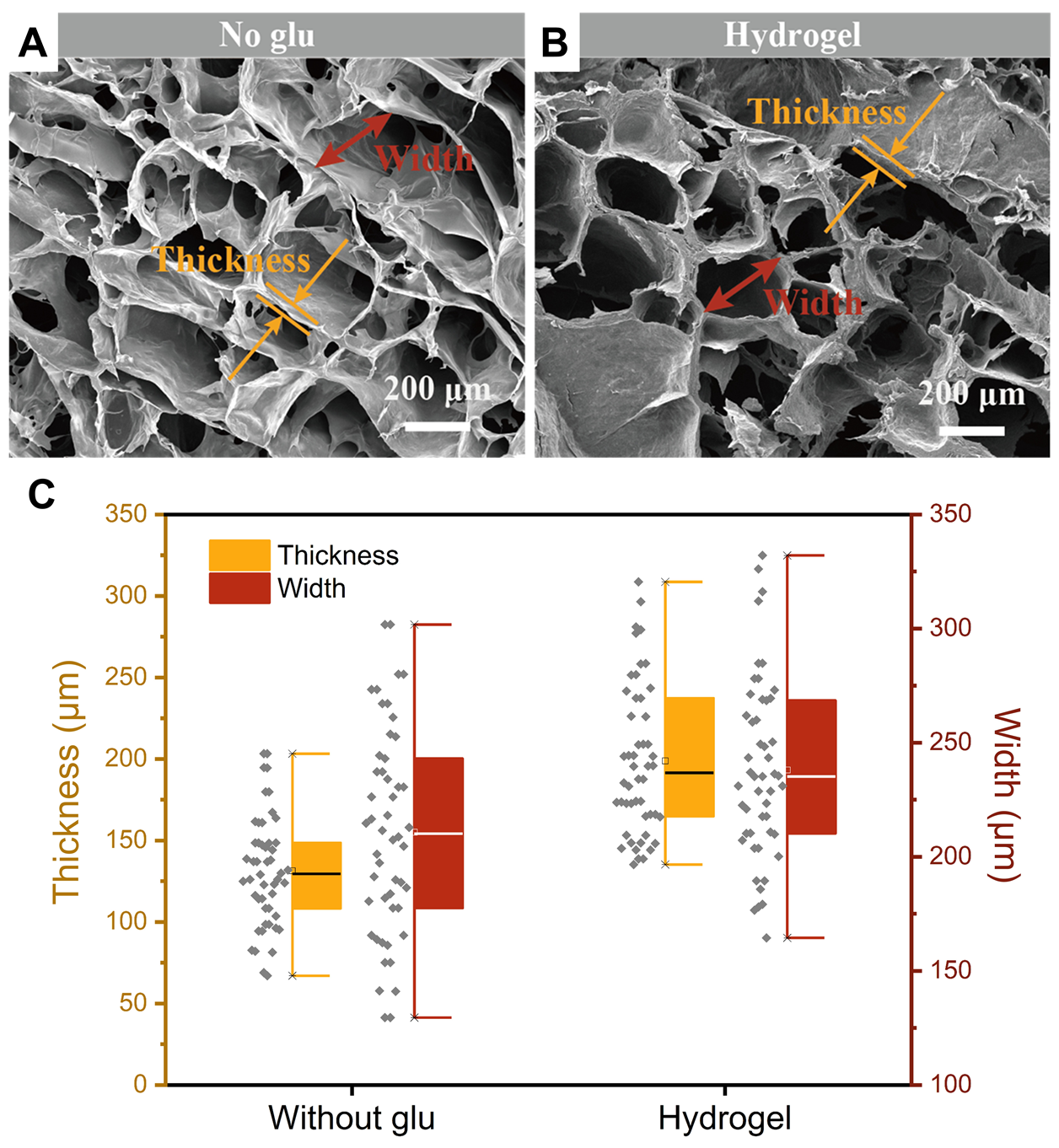
**

**Fig. S13** Scanning electron microscopy (SEM) images of hydrogels prepared with **A** and without **B** glutaraldehyde crosslinking, accompanied by statistical analysis **C** of the pore size distribution derived from image-based quantification

**
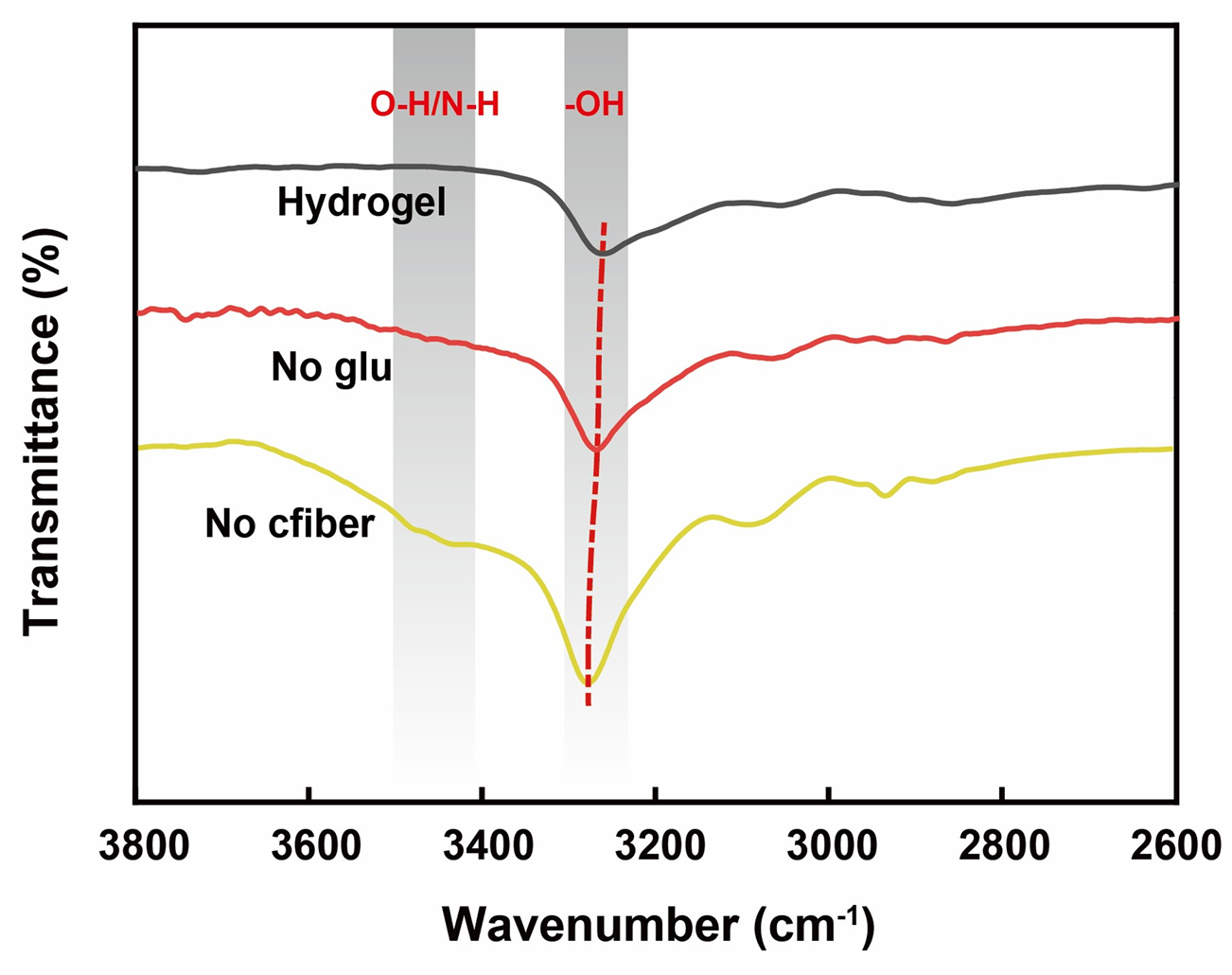
**

**Fig. S14** FTIR spectra of the highly absorbent hydrogel. The broad absorption band around 3400 cm^−1^ corresponds to overlapping O–H and N–H stretching vibrations, while the shoulder near 3300 cm^−1^ is primarily attributed to free O–H stretching


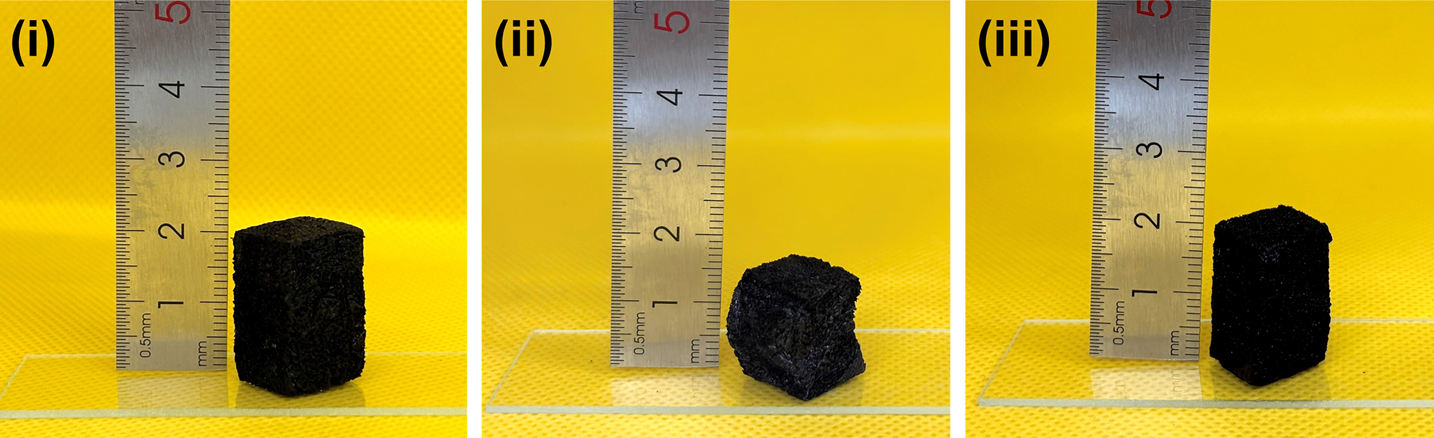


**Fig. S15** Photographic images showing the compressibility and shape recovery of the highly absorbent hydrogel under 90% strain: (i) Before compression, (ii) immediately removed after 90% compression, (iii) after 10 s immersion


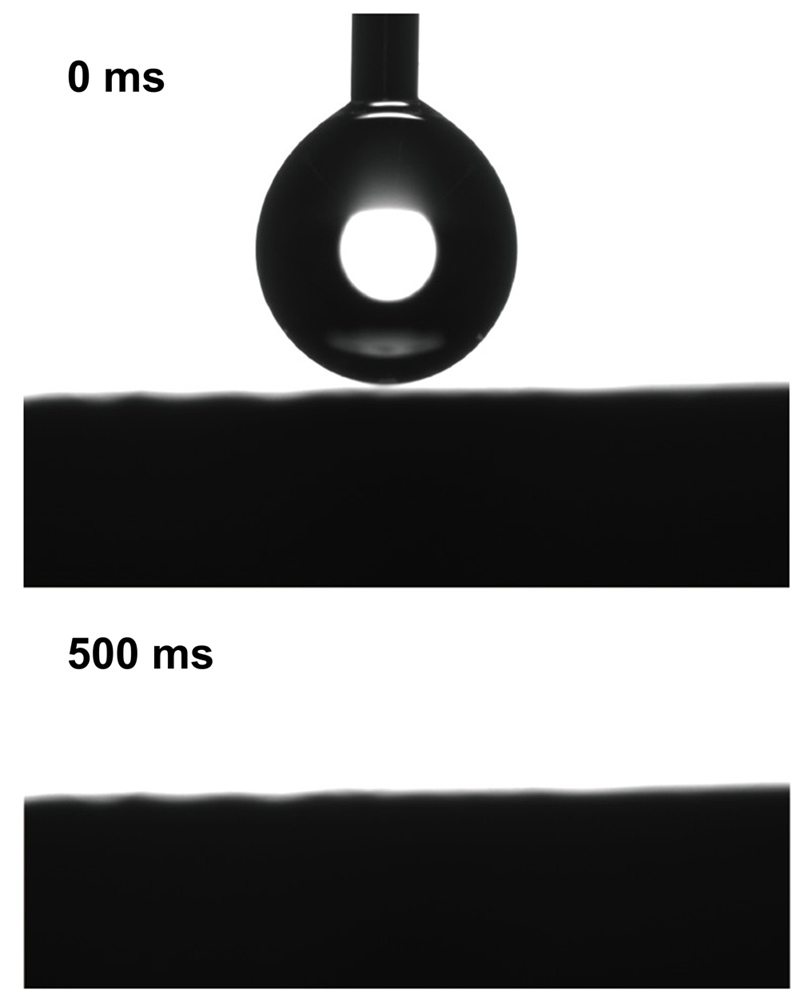


**Fig. S16** Water contact angle (WCA) measurement of the chitin nanofiber-based hydrogel. Upon contact, the water droplet was instantly absorbed into the porous matrix, indicating a superhydrophilic surface with a contact angle approaching 0°. The test was conducted at room temperature using a standard sessile drop method with deionised water


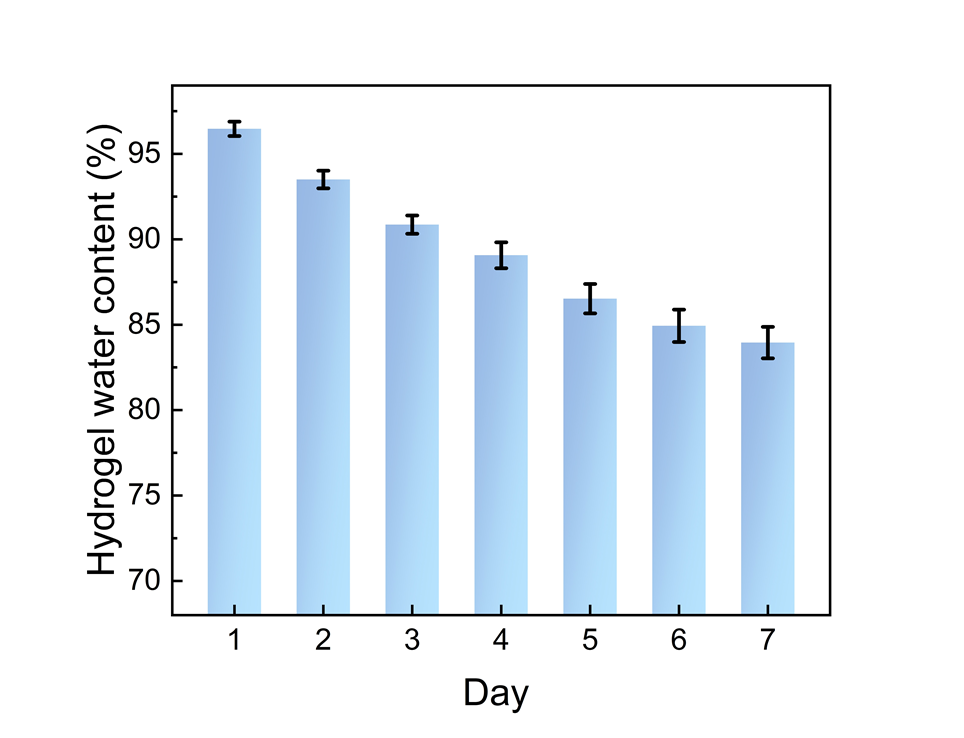


**Fig. S17** Long-term water retention performance of the hydrogel


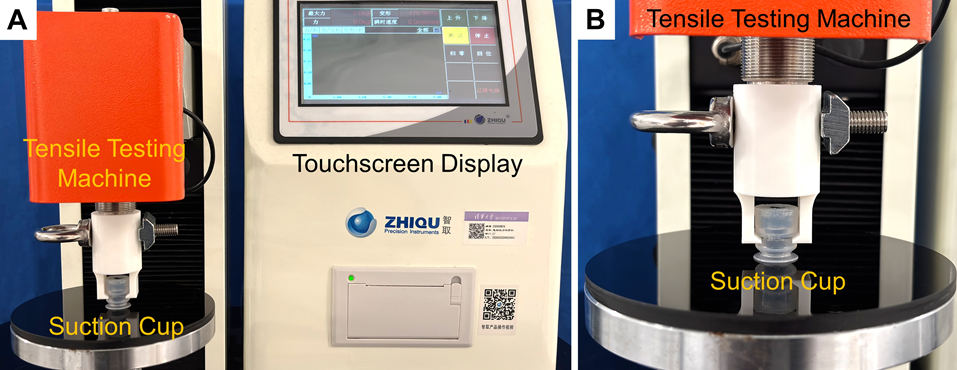


**Fig. S18** **A** Photograph of the adsorption force testing experiment. **B** Local enlarged image of the experiment


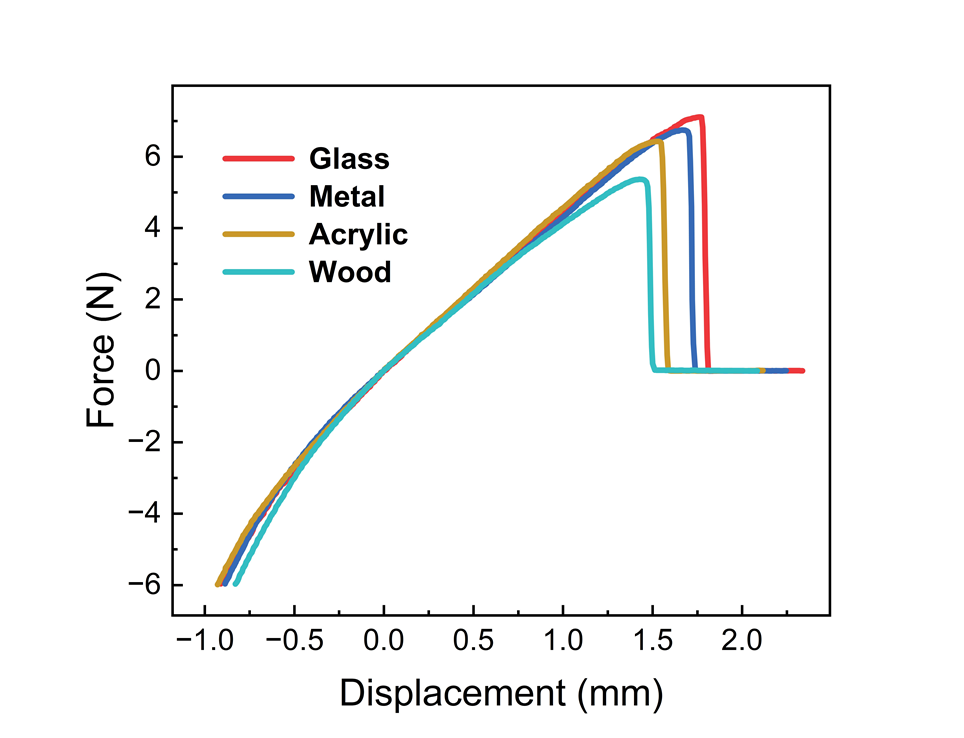


**Fig. S19** Force–displacement curves of the UHSVS tested on different material surfaces


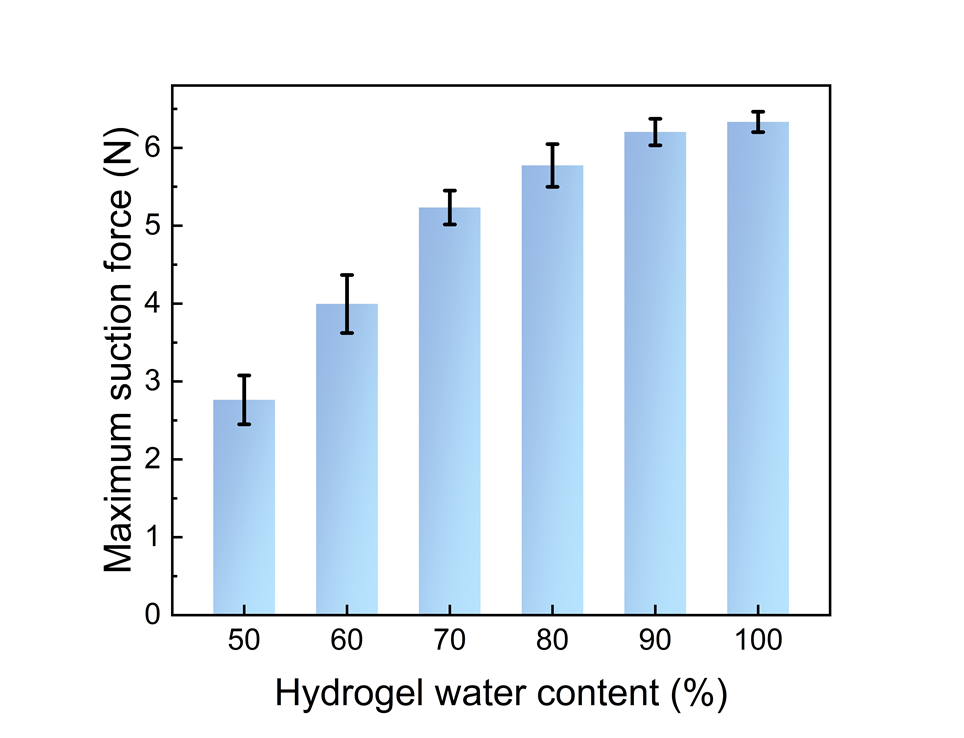


**Fig. S20** The impact of hydrogel water content on suction force


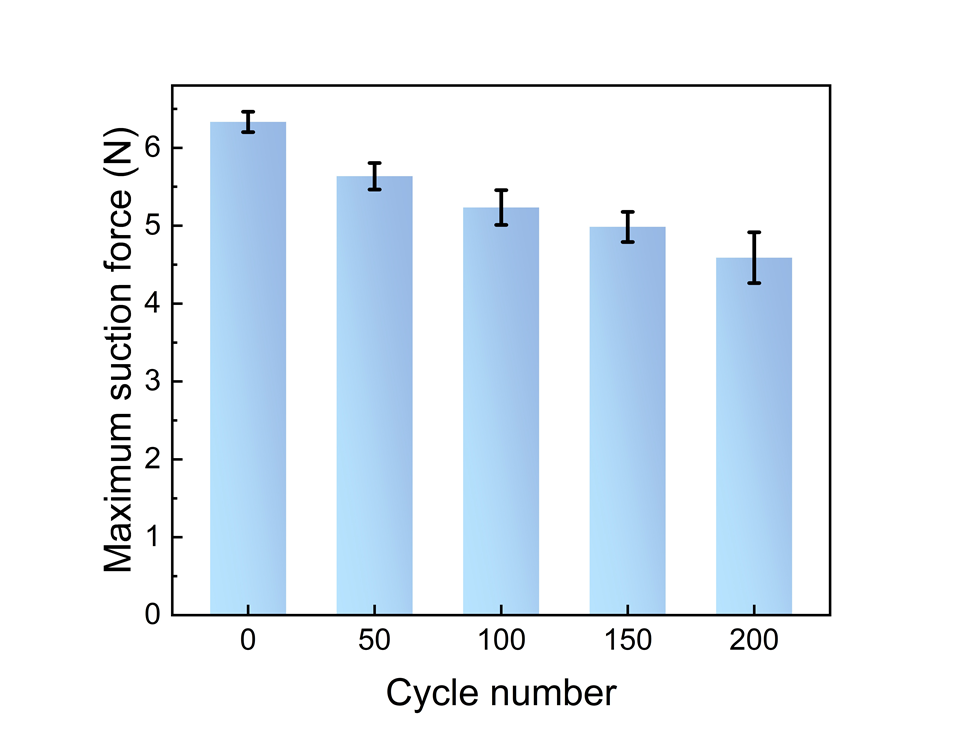


**Fig. S****21** Suction force versus number of suction cycles (0-200) for hydrogel with water and PBP-silicone suction cup configurations


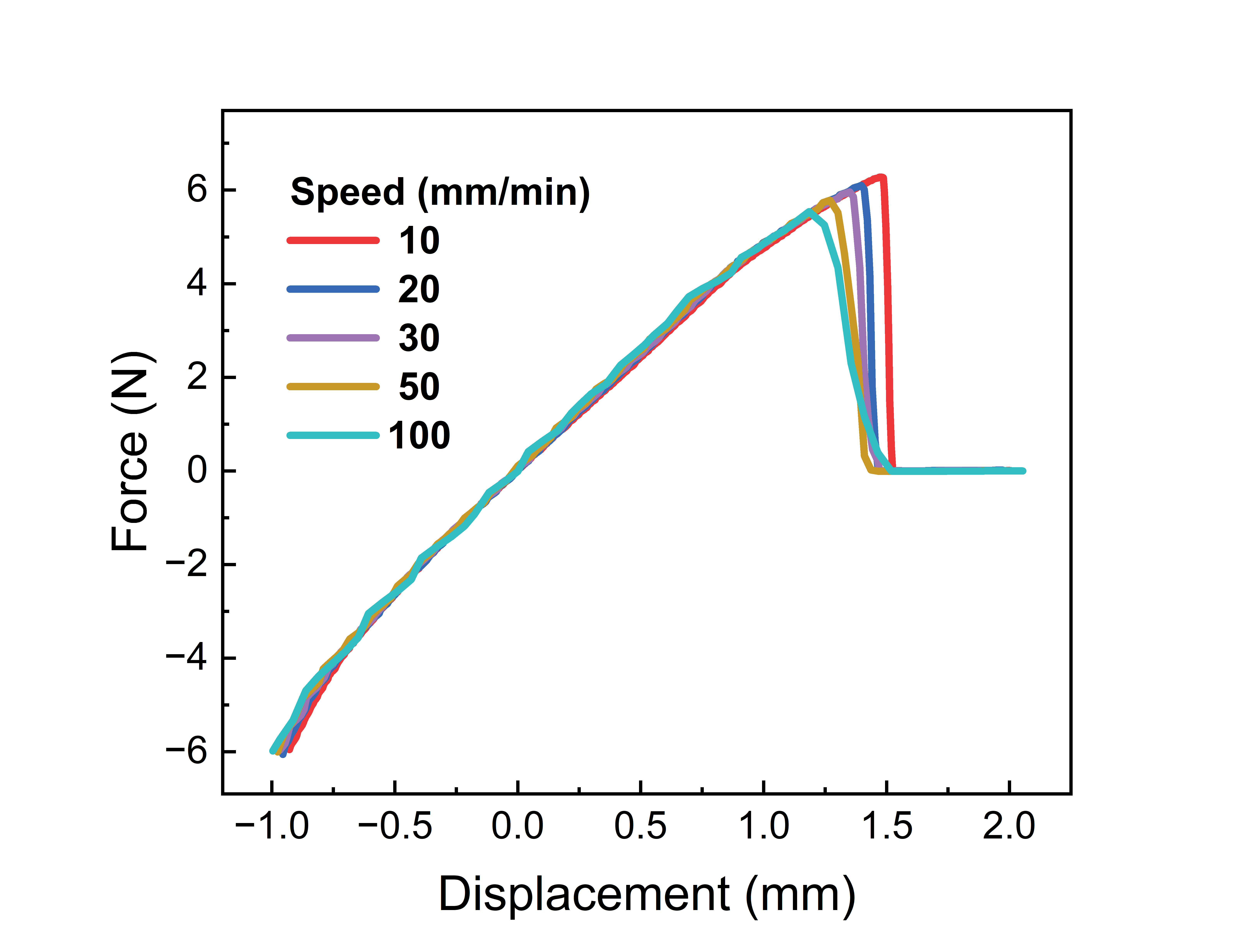


**Fig. S22** Suction force-displacement process at different pull-off speeds


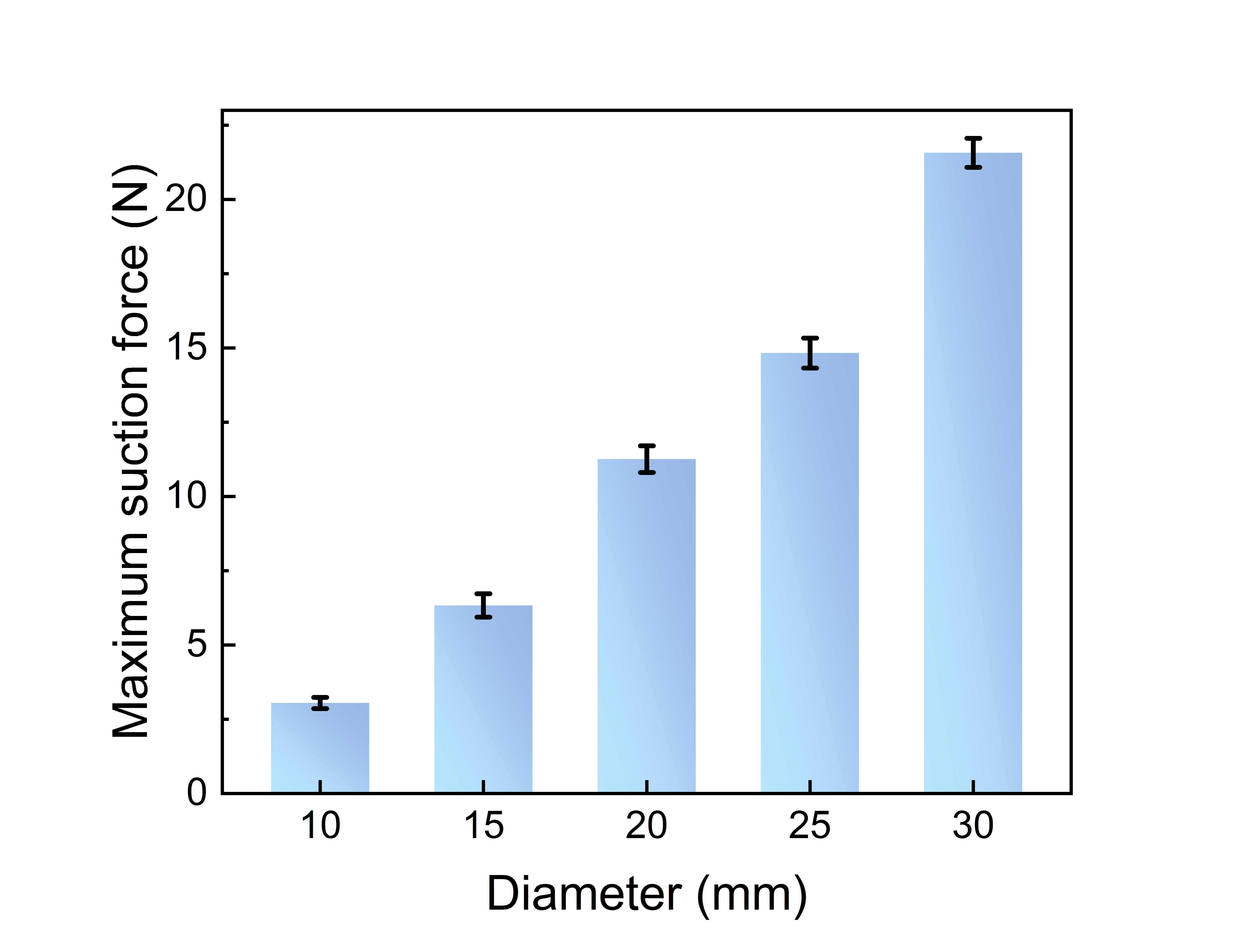


**Fig. S23** The impact of suction cup diameter on suction force


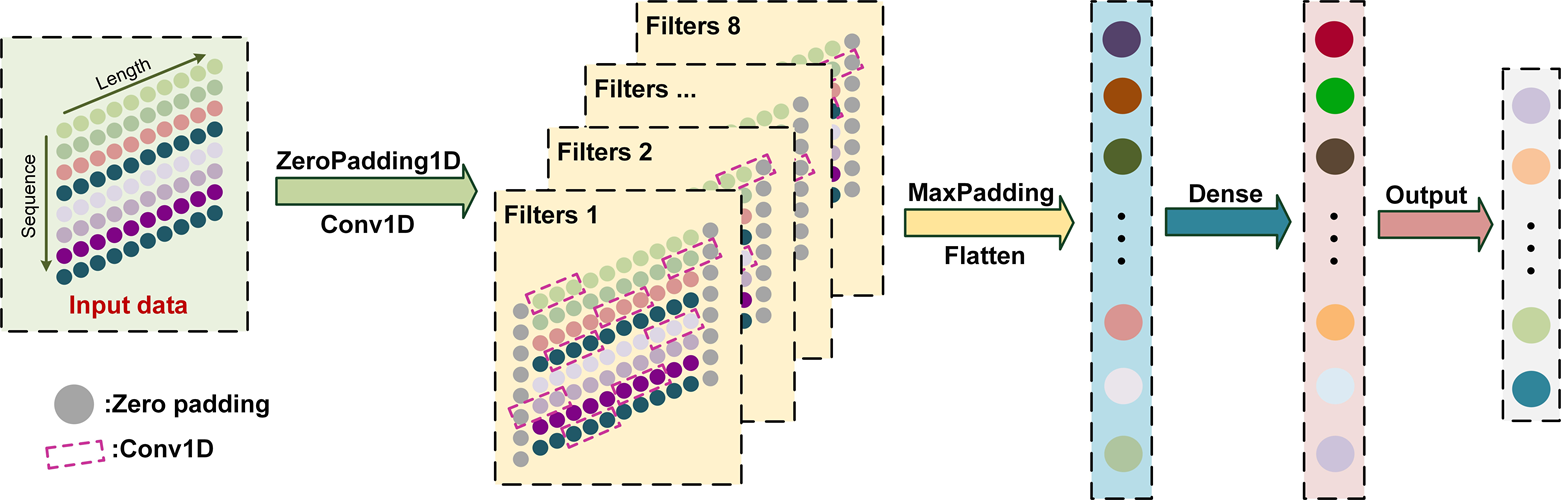


**Fig. S24** The architecture of the network used for sensor unit data processing

**
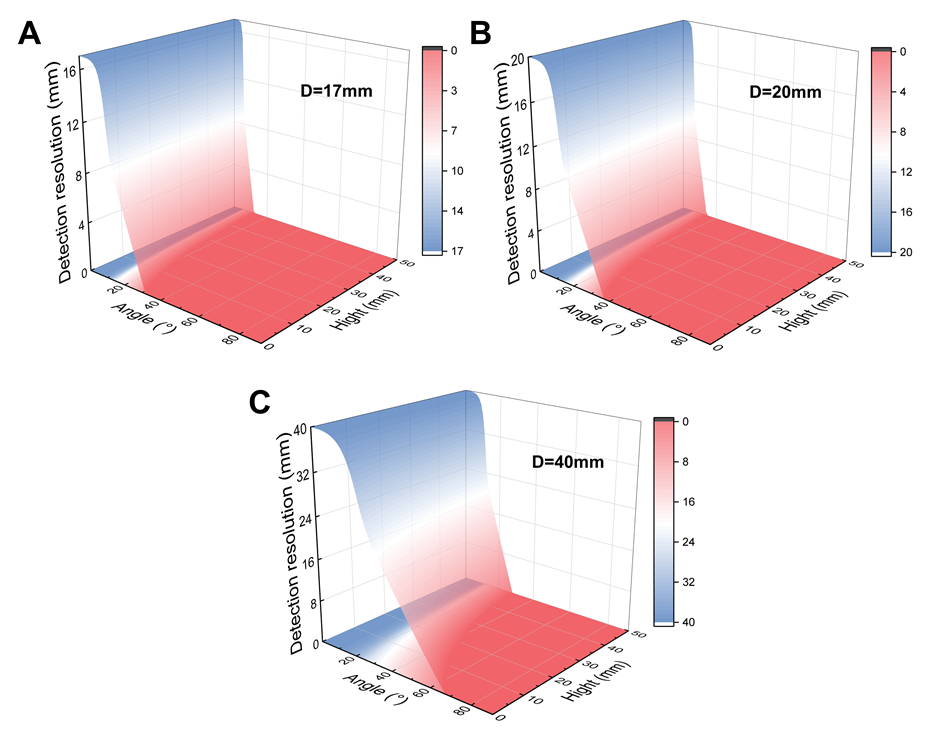
**

**Fig. S25** Influence of angle and height on detection resolution of the sensor units **A** D=17 mm, **B** D =20 mm, **C** D =40 mm

**Supplementary References**

1. Q. Hu, E. Dong, G. Cheng, H. Jin, J. Yang et al., Inchworm-inspired soft climbing robot using microspine arrays. 2019 IEEE/RSJ International Conference on Intelligent Robots and Systems (IROS). pp. 5800–5805. IEEE (2020). <https://doi.org/10.1109/IROS40897.2019.8967601>
2. E.W. Hawkes, D.L. Christensen, M.R. Cutkosky, Vertical dry adhesive climbing with a 100 × bodyweight payload. 2015 IEEE International Conference on Robotics and Automation (ICRA). pp. 3762–3769. IEEE (2015). <https://doi.org/10.1109/ICRA.2015.7139722>
3. G. Gu, J. Zou, R. Zhao, X. Zhao, X. Zhu, Soft wall-climbing robots. Sci. Robot. **3**(25), eaat2874 (2018). <https://doi.org/10.1126/scirobotics.aat2874>
4. B. Liao, H. Zang, M. Chen, Y. Wang, X. Lang et al., Soft rod-climbing robot inspired by winding locomotion of snake. Soft Robot. **7**(4), 500–511 (2020). <https://doi.org/10.1089/soro.2019.0070>
5. M.A. Robertson, J. Paik, New soft robots really suck: Vacuum-powered systems empower diverse capabilities. Sci. Robot. **2**(9), eaan6357 (2017). <https://doi.org/10.1126/scirobotics.aan6357>
6. X. Liu, K. Li, S. Qian, L. Niu, W. Chen et al., A high-sensitivity flexible bionic tentacle sensor for multidimensional force sensing and autonomous obstacle avoidance applications. Microsyst. Nanoeng. **10**(1), 149 (2024). <https://doi.org/10.1038/s41378-024-00749-7>
7. N.A. Ahmad Ridzuan, N. Miki, Tooth-inspired tactile sensor for detection of multidirectional force. Micromachines **10**(1), 18 (2018). <https://doi.org/10.3390/mi10010018>
8. J.T. Reeder, T. Kang, S. Rains, W. Voit, 3D, reconfigurable, multimodal electronic whiskers *via* directed air assembly. Adv. Mater. **30**(11), 1706733 (2018). <https://doi.org/10.1002/adma.201706733>
9. S. Kim, C. Velez, D.K. Patel, S. Bergbreiter, A magnetically transduced whisker for angular displacement and moment sensing. 2019 IEEE/RSJ International Conference on Intelligent Robots and Systems (IROS). pp. 665–671. IEEE (2020). <https://doi.org/10.1109/IROS40897.2019.8968518>
10. H. Zhang, L. Weng, G. Lin, Z. Li, S. Jiang et al., Development and application of magnetic tentacles array for sensing tiny forces. Measurement **240**, 115533 (2025). <https://doi.org/10.1016/j.measurement.2024.115533>
11. C. Chen, X.-L. Li, S. Zhao, Y. Song, Y. Zhu et al., A biomimetic e-whisker sensor with multimodal perception and stimuli discrimination. Device **1**(5), 100148 (2023). <https://doi.org/10.1016/j.device.2023.100148>
12. C. Jiang, H. Xu, L. Yang, J. Liu, Y. Li et al., Neuromorphic antennal sensory system. Nat. Commun. **15**, 2109 (2024). <https://doi.org/10.1038/s41467-024-46393-7>
13. D. Zhu, J. Lu, M. Zheng, D. Wang, J. Wang et al., Self-powered bionic antenna based on triboelectric nanogenerator for micro-robotic tactile sensing. Nano Energy **114**, 108644 (2023). <https://doi.org/10.1016/j.nanoen.2023.108644>
14. H. Varghese, V. Priya K, U.N.S. Hareesh, A. Chandran, Nanofibrous PAN-PDMS films-based high-performance triboelectric artificial whisker for self-powered obstacle detection. Macromol. Rapid Commun. **45**(2), 2300462 (2024). <https://doi.org/10.1002/marc.202300462>
15. J. An, P. Chen, Z. Wang, A. Berbille, H. Pang et al., Biomimetic hairy whiskers for robotic skin tactility. Adv. Mater. **33**(24), e2101891 (2021). <https://doi.org/10.1002/adma.202101891>
16. P. Xu, X. Wang, S. Wang, T. Chen, J. Liu et al., A triboelectric-based artificial whisker for reactive obstacle avoidance and local mapping. Research **2021**, 9864967 (2021). <https://doi.org/10.34133/2021/9864967>
17. J. Liu, P. Xu, J. Zheng, X. Liu, X. Wang et al., Whisker-inspired and self-powered triboelectric sensor for underwater obstacle detection and collision avoidance. Nano Energy **101**, 107633 (2022). <https://doi.org/10.1016/j.nanoen.2022.107633>
18. S. Wang, P. Xu, X. Wang, J. Zheng, X. Liu et al., Underwater bionic whisker sensor based on triboelectric nanogenerator for passive *Vortex* perception. Nano Energy **97**, 107210 (2022). <https://doi.org/10.1016/j.nanoen.2022.107210>
19. X. Hou, L. Xin, Y. Fu, Z. Na, G. Gao et al., A self-powered biomimetic mouse whisker sensor (BMWS) aiming at terrestrial and space objects perception. Nano Energy **118**, 109034 (2023). <https://doi.org/10.1016/j.nanoen.2023.109034>
20. M. Wu, H. Geng, Y. Hu, H. Ma, C. Yang et al., Superelastic graphene aerogel-based metamaterials. Nat. Commun. **13**(1), 4561 (2022). <https://doi.org/10.1038/s41467-022-32200-8>
21. H. Geng, B. Yao, J. Zhou, K. Liu, G. Bai et al., Size fractionation of graphene oxide nanosheets *via* controlled directional freezing. J. Am. Chem. Soc. **139**(36), 12517–12523 (2017). <https://doi.org/10.1021/jacs.7b05490>
22. Y. Guo, Y. Han, Y. Cao, Y. Chen, J. Xie et al., Facile fabrication of tough super macroporous hydrogel *via* enhanced phase separation. Adv. Funct. Mater. **35**(2), 2412412 (2025). <https://doi.org/10.1002/adfm.202412412>
23. W. Lu, X. Li, Y. Wang, F. Yao, X. Wang et al., All-regional highly efficient moisture capturing and sunlight driven steam generation. Water Res. **279**, 123398 (2025). <https://doi.org/10.1016/j.watres.2025.123398>
24. X. Li, P. Wang, Q. Lu, H. Yao, C. Yang et al., A hierarchical porous aerohydrogel for enhanced water evaporation. Water Res. **244**, 120447 (2023). <https://doi.org/10.1016/j.watres.2023.120447>
25. H. Geng, C. Lv, M. Wu, H. Ma, H. Cheng et al., Biomimetic antigravity water transport and remote harvesting powered by sunlight. Glob. Chall. **4**(11), 2000043 (2020). <https://doi.org/10.1002/gch2.202000043>
26. T. Yue, H. Bloomfield-Gadêlha, J. Rossiter, Snail-inspired water-enhanced soft sliding suction for climbing robots. Nat. Commun. **15**(1), 4038 (2024). <https://doi.org/10.1038/s41467-024-48293-2>
27. M. Yao, J. Fang, Hydrophilic PEO-PDMS for microfluidic applications. J. Micromech. Microeng. **22**(2), 025012 (2012). <https://doi.org/10.1088/0960-1317/22/2/025012>
